# A crystal growth kinetics guided Cu aerogel for highly efficient CO_2_ electrolysis to C_2+_ alcohols†

**DOI:** 10.1039/d2sc04961a

**Published:** 2022-12-06

**Authors:** Pengsong Li, Jiahui Bi, Jiyuan Liu, Qinggong Zhu, Chunjun Chen, Xiaofu Sun, Jianling Zhang, Zhimin Liu, Buxing Han

**Affiliations:** a Beijing National Laboratory for Molecular Sciences, CAS Key Laboratory of Colloid, Interface and Chemical Thermodynamics, CAS Research/Education Center for Excellence in Molecular Sciences, Institute of Chemistry, Chinese Academy of Sciences Beijing 100190 P. R. China qgzhu@iccas.ac.cn hanbx@iccas.ac.cn; b University of Chinese Academy of Sciences Beijing 100049 P. R. China; c Shanghai Key Laboratory of Green Chemistry and Chemical Processes, School of Chemistry and Molecular Engineering, East China Normal University Shanghai 200062 P. R. China; d Institute of Eco-Chongming 20 Cuiniao Road, Chenjia Town, Chongming District Shanghai 202162 P. R. China

## Abstract

To realize commercial CO_2_ electrochemical reduction to C_2+_ alcohols, the selectivity and production rate should be further improved. Establishing controllable surface sites with a favorable local environment is an interesting route to guide the C_2+_ pathway. Herein, we report a room-temperature one-step synthetic strategy to fabricate a highly stable Cu aerogel as an efficient CO_2_ reduction electrocatalyst. Controlling crystal growth kinetics using different reductants is an efficient strategy to modulate the nucleation and growth rate of Cu aerogels, enabling the creation of efficient surface sites for the C_2+_ pathway. Over the Cu aerogel obtained by reducing Cu^2+^ using a weak reductant (NH_3_·BH_3_), the faradaic efficiency of C_2+_ products could reach 85.8% with the current density of 800 mA cm^−2^ at the potential of −0.91 V *vs.* reversible hydrogen electrode, and the C_2+_ alcohol selectivity was 49.7% with a partial current density of 397.6 mA cm^−2^, while the Cu aerogel prepared using a strong reductant (NaBH_4_) was favorable to generating CO. Experimental and theoretical studies showed that the selectivity of the reaction depended strongly on the desorption and dimerization of *CO intermediates on the catalysts. The strong reductant induced a defective Cu surface that could facilitate the desorption of the *CO intermediate, subsequently producing CO, whereas the low defect Cu produced using a weak reductant could significantly enhance the selectivity for the C_2+_ product by improving *CO adsorption and the C–C coupling on the catalyst. This work opens a new way for constructing efficient electrocatalysts for CO_2_ electroreduction to C_2+_ alcohols.

## Abstract 2

Controlling crystal growth kinetics using different reductants is an efficient strategy to modulate the surficial chemistry micro-circumstance of Cu aerogels, enabling the creation of efficient surface sites for increasing C_2+_ product selectivity.

## Graphical abstract

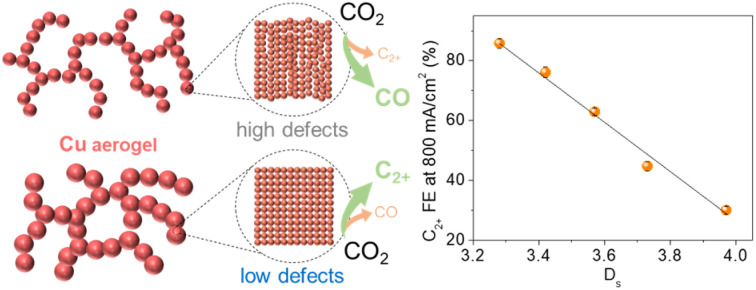

## Introduction

Electrochemical conversion of CO_2_ is a promising strategy to abate the consumption of fossil resources and close the carbon neutral energy cycle.^1,2^ The reduction products, such as high-energy density multi-carbon (C_2+_) alcohols, are of particular interest, as they have a correspondingly high market price consistent with global demands.^3^ To date, many electrocatalysts have been designed to improve the catalytic performance of the CO_2_ reduction reaction (CO_2_RR) to C_2+_ alcohols, such as FeTPP[Cl]/Cu (5,10,15,20-tetraphenyl-21*H*,23*H*-porphine iron(iii) chloride/Cu),^4^ N–C/Cu (nitrogen-doped carbon layer on Cu),^5^ Cu–CuI,^6^ Cu_2_S–Cu–vacancy^7^ and Ce(OH)_*X*_/Cu.^8^ Remarkably, the CO_2_RR to ethanol has been reported using an N–C/Cu catalyst with a faradaic efficiency (FE) of up to 53.7% with a partial current density of 161.1 mA cm^−2^.^5^ Over the Cu–CuI catalyst, the optimum partial current density of C_2+_ alcohols could reach 299 mA cm^−2^ with a moderate selectivity of 33.4%.^6^ Several catalyst design strategies have also been developed,^9–11^ including precipitation, electrodeposition, sputtering, evaporation, defect engineering, hybridization, surface modifications and reconstructions. However, the CO_2_ to C_2+_ alcohol electrocatalytic system still remains limited by the low productivity rates at high product selectivity, making it difficult to meet the commercial demand.^12,13^ This is due to the complex and uncontrollable reaction pathways during the CO_2_ reduction.^3^ The binding environment of the *CO intermediate on the active sites determines the subsequent reaction paths.^14,15^ The *CO intermediate can either undergo desorption to release the CO product or dimerization to form *OCCO embarking on the C_2+_ pathway.^16–19^ Therefore, it is vital to develop highly active surface sites that improve the *CO adsorption properties and ultimately promote the C–C coupling to form C_2+_ alcohols.^20,21^

Tuning the surface structure at the atomic level is of primary importance in the generation of efficient active sites for the CO_2_RR.^22,23^ At the nanoscale, crystal defects largely influence the properties and functionalities of catalysts.^24–26^ Specifically, each active site has its own features due to the unique local structural environment, and the catalytic activity towards the CO_2_RR can be modified by the presence of surface defects.^27,28^ It has been reported that creating defects can enhance the CO_2_RR performance, but the specific defect–activity relationships have not been fully elucidated.^29,30^ That is, structural disorder is not detrimental, but may not be all beneficial to the C_2+_ pathway. Considering the high complexity of CO_2_ reduction toward C_2+_ products, the major bottleneck in the catalyst design lies in establishing controllable surface sites and, ultimately, guiding the reaction pathway.^31^

Here, we demonstrate a room-temperature one-step synthetic strategy to fabricate a highly stable Cu aerogel as an efficient CO_2_RR electrocatalyst. Controlling crystal growth kinetics can efficiently modulate the nucleation and growth rate of Cu aerogels, enabling the creation of efficient surface sites for increasing C_2+_ product selectivity. Theoretical studies suggest that the adsorption strength of *CO intermediates on Cu sites depends strongly on the defects. By reducing the defect level on the Cu aerogel surface, the desorption and dimerization of *CO intermediates can be regulated to a preferable C_2+_ pathway. A strong reductant (sodium borohydride, NaBH_4_) induces abundant irregular defects in the Cu aerogel to facilitate the desorption of the *CO intermediate, subsequently producing CO, while the low defect Cu produced under weak reducing conditions (borane ammonia complex, NH_3_·BH_3_) can easily facilitate the C–C coupling pathway, which leads to C_2+_ products. Remarkably, the Cu aerogel obtained by reducing Cu^2+^ using a weak reductant (NH_3_·BH_3_) can yield a C_2+_ FE of 85.8% at the current density of 800 mA cm^−2^ and the potential of −0.91 V *vs.* reversible hydrogen electrode (RHE), and the C_2+_ alcohol selectivity was 49.7% with a partial current density of 397.6 mA cm^−2^.

## Results and discussion

**Fig. 1:**
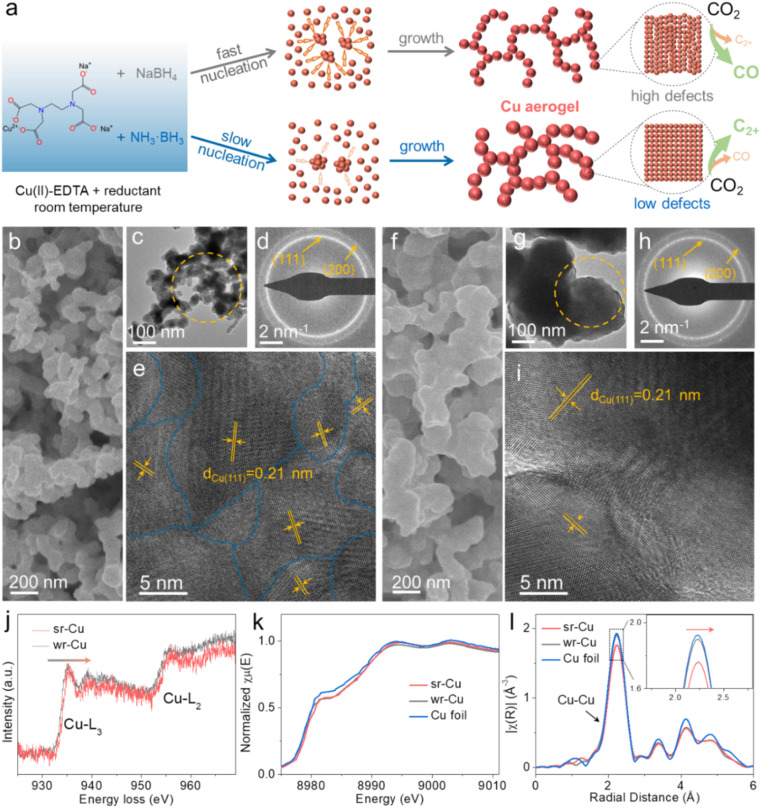
(a) Schematic diagram of the formation of the Cu aerogels with high defects (sr-Cu) and low defects (wr-Cu) at room temperature. (b) SEM, (c) TEM, (d) SAED patterns and (e) HRTEM images of the sr-Cu aerogel. (f) SEM, (g) TEM, (h) SAED patterns and (i) HRTEM images of the wr-Cu aerogel. (j) The EELS profiles of the Cu-L_2,3_ edge recorded across the sr-Cu and wr-Cu aerogels. (k) The XANES spectra and (l) Fourier-transform Cu K-edge EXAFS spectra of sr-Cu, wr-Cu aerogels and Cu foil.

Our study focused on modulating the nucleation and growth rate of Cu to change the surface structure of Cu aerogels. In the chemical reduction process, Cu^2+^ in the solution was reduced to metallic Cu^0^ and then it agglomerated into clusters growing up into an aerogel at room temperature. We selected reductants with different reduction reactivities to control the crystal growth kinetics in the process. Using a strong reductant (NaBH_4_), the Cu nucleation proceeds rapidly, leading to a small particle size and an irregular defect-rich surface, whereas the crystal nucleation rate in the weak reductant (NH_3_·BH_3_) solution is slower than that in the strong reductant solution, resulting in a larger particle size and low defects (Fig. 1a). The as-synthesized Cu aerogels are denoted as sr-Cu (strong reductant-induced Cu) and wr-Cu (weak reductant-induced Cu). In order to establish trends in crystal growth rates with respect to the defect level, we prepared a series of Cu aerogels using reductant mixtures of NaBH_4_ and NH_3_·BH_3_ with different compositions (the molar ratios of NaBH_4_ : NH_3_·BH_3_ = 5 : 1, 1 : 1 and 1 : 5).

Scanning electron microscopy (SEM) and transmission electron microscopy (TEM) images showed that both sr-Cu and wr-Cu had a porous network structure self-assembled by nanoparticles (Fig. 1b, c, f and g) and demonstrated that the particle size of Cu became larger when applying a weaker reductant. Furthermore, the surface areas of sr-Cu and wr-Cu determined by the Brunauer–Emmett–Teller method were 10.53 and 5.75 m^2^ g^−1^, respectively. Fig. 1d and h show the selected area electron diffraction (SAED) patterns of sr-Cu and wr-Cu particles marked in Fig. 1c and g, respectively. They reveal that sr-Cu and wr-Cu had a well-defined cubic crystal phase with diffraction rings indexed to (111) and (200) lattices. High-resolution transmission electron microscopy (HRTEM) images confirmed that the lattice spacings of both sr-Cu and wr-Cu aerogels were 0.21 nm, corresponding to the lattice plane distance of the (111) plane of face-centered cubic metallic Cu. The main difference was that the wr-Cu crystal surface (Fig. 1i) was flatter than the sr-Cu crystal surface (Fig. 1e), which indicates lower defects in wr-Cu. The obvious trend of 5 : 1-Cu to 1 : 5-Cu further confirmed that a weak reductant could give rise to lower crystal surface defects than a strong reductant (Fig S1–S3†). The electron energy loss spectra (EELS) of sr-Cu and wr-Cu were also recorded. As shown in Fig. 1j, it is observed that the two main features of these edges are the strong white-lines L_3_ and L_2_ separated by about 20 eV, which is attributed to the spin orbit splitting of the 2p core hole consistent with the metallic Cu.^32^ Furthermore, compared with wr-Cu, the Cu-L_3_ edge of sr-Cu was shifted by 0.3 eV towards the higher energy region. The Cu K-edge X-ray absorption near edge structure (XANES) (Fig. 1k) clearly revealed that the Cu K edge position (8979.04 eV) of wr-Cu was similar to that of metallic Cu (8979.01 eV), while the Cu K-edge position of sr-Cu was located at a higher energy (8979.31 eV). The Fourier-transform extended X-ray absorption fine structure (EXAFS) spectrum can accurately reveal the local structure of a catalyst.^33^ When comparing with Cu metal and wr-Cu, sr-Cu possessed a slightly longer first-shell Cu–Cu bond (Fig. 1l). In addition, the small angle X-ray scattering (SAXS) technique was employed to analyze the fractal structure. The surface fractal (*D*_s_) values were obtained from the ln(*I*(*h*)) *vs.* ln(*h*) plots (Fig. S4†). It can be seen that the surface of sr-Cu (*D*_s_ = 3.97) was coarser than that of wr-Cu (*D*_s_ = 3.28), indicating the existence of more defects in the Cu catalyst obtained using a strong reductant.

Powder X-ray diffraction (XRD) data demonstrated the presence of metallic Cu (PDF#04-0836) in these Cu aerogels (Fig. S5†). Among them, sr-Cu had a weak-intensity (111) diffraction peak, indicating its low crystallinity. The elemental valence states and chemical composition of the Cu aerogel surfaces were also investigated by X-ray photoelectron spectroscopy (XPS). For all the Cu aerogels, two main peaks around 952.1 eV and 932.3 eV were observed (Fig. S6a†), corresponding to Cu 2p_1/2_ and Cu 2p_3/2_ peaks, respectively. Auger electron spectroscopy (AES) further confirmed that the Cu species were composed of Cu^0^ and Cu^+^ (Fig. S6b†) on the surface.^34^ The peak located at 570 eV (Cu^+^) was attributed to the surface oxidation of metallic Cu in air after taking it out from the 1 M KOH electrolyte. The N 1s and B 1s spectra in Fig. S7† suggest that the aerogels were free from N and B elements. In addition, no N or B was detected in the inductively coupled plasma mass spectrometry (ICP-MS) measurement over the wr-Cu and sr-Cu aerogels. Combining the above results, we can conclude that the as-synthesized aerogel catalysts are mainly metallic Cu.

**Fig. 2:**
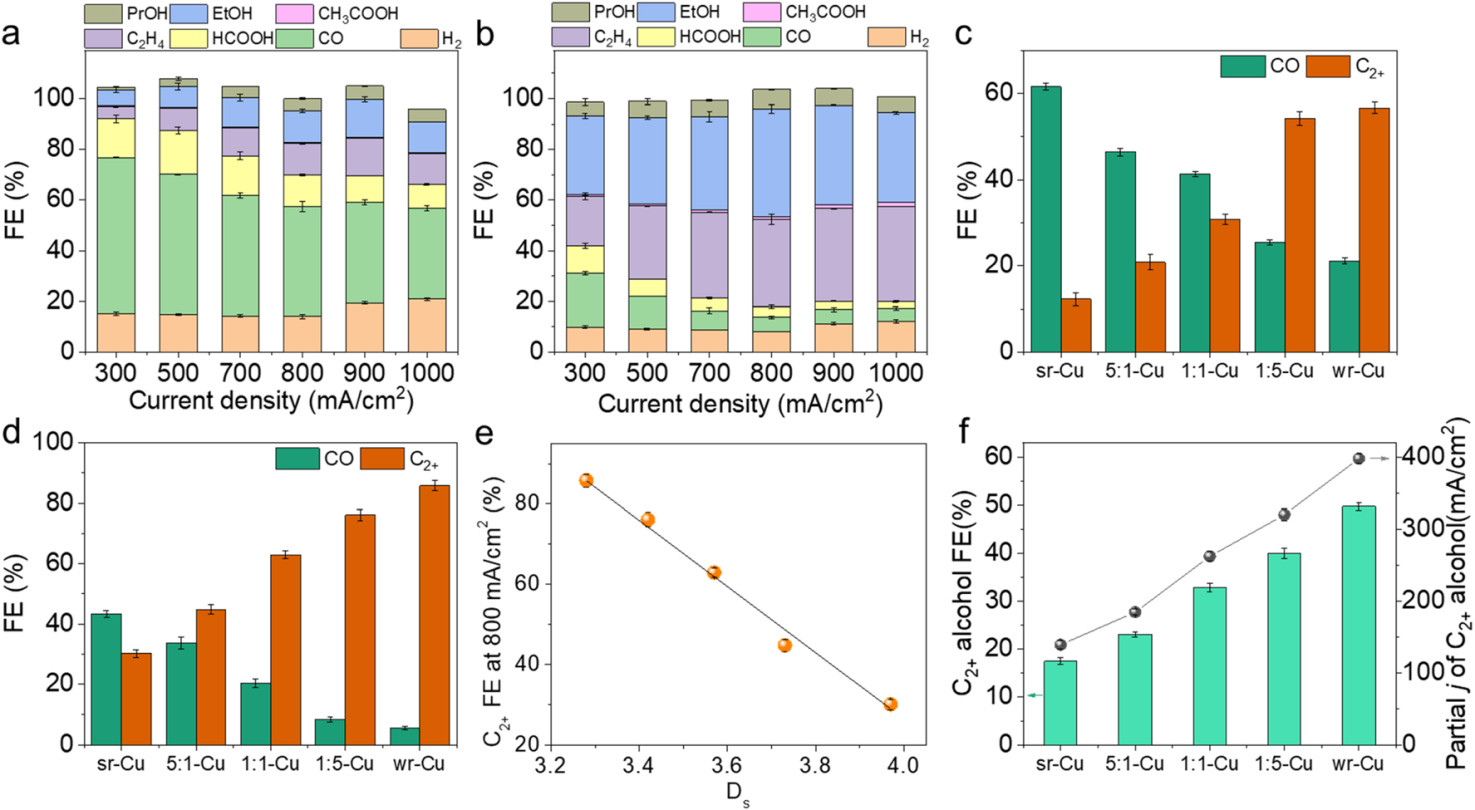
FE for each CO_2_RR product and H_2_ on (a) sr-Cu and (b) wr-Cu at various current densities ranging from 300 to 1000 mA cm^−2^. Error bars represent the standard deviations from multiple measurements. CO and C_2+_ FE values on different catalysts under the current densities of (c) 300 mA cm^−2^ and (d) 800 mA cm^−2^. (e) Plot of *D*_s_ values *vs.* FE of C_2+_ products over sr-Cu, 5 : 1-Cu, 1 : 1-Cu, 1 : 5-Cu and wr-Cu at the current density of 800 mA cm^−2^. (f) FE and partial current density of C_2+_ alcohols on different catalysts at the current densities of 800 mA cm^−2^.

The as-prepared Cu aerogels were used as electrocatalysts in a flow cell reactor using 1 M KOH solution as the electrolyte to evaluate the CO_2_ electrochemical reduction performance. The wr-Cu catalyst resulted in a larger total current density at the same potential than the sr-Cu, 5 : 1-Cu, 1 : 1-Cu and 1 : 5-Cu catalysts with the CO_2_ feed gas, suggesting its high electrocatalytic activity (Fig. S8 and S9†). Under the reaction conditions, the liquid products HCOOH, CH_3_COOH, CH_3_CH_2_OH (EtOH), and CH_3_CH_2_CH_2_OH (PrOH) were detected by ^1^H nuclear magnetic resonance (^1^H-NMR) (Fig. S10†), and H_2_, CO, and C_2_H_4_ were the gaseous products determined using gas chromatography. As displayed in Fig. 2a, b and S11,† all the Cu aerogels yielded products with a combined FE of around 100%. As the major C_1_ product, the FE of CO was gradually suppressed with increasing current density, and the selectivity of C_2+_ products was enhanced simultaneously. Taking the electrolysis at 300 mA cm^−2^ and 800 mA cm^−2^ as examples, sr-Cu delivered the highest CO FE and wr-Cu exhibited the highest C_2+_ FE, suggesting that the *CO desorption occurred easily on the crystal surface of sr-Cu, but it was significantly suppressed on wr-Cu. The other catalysts had properties somewhat between sr-Cu and wr-Cu, and the CO selectivity followed an increasing sequence of wr-Cu < 1 : 5-Cu < 1 : 1-Cu < 5 : 1-Cu < sr-Cu. This phenomenon of increased FE of CO was also observed on the OD Cu catalyst with high roughness.^35^ As expected, the FE values of C_2+_ products showed the opposite sequence (Fig. 2c and d). These differences suggest that the CO product formation was inhibited, and the generation of C_2+_ products was promoted by increasing the crystal surface defect of the Cu aerogel. We correlated the *D*_s_ values with the FE of C_2+_ products and found a linear relationship (Fig. 2e) indicating that the defect level in the catalysts is directly related to the performance of the electrochemical reduction of CO_2_ to C_2+_ products. Especially, using wr-Cu as the catalyst, the FE of total C_2+_ products could be as high as 85.8% with the current density of 800 mA cm^−2^ at a low applied potential of −0.91 V *vs.* RHE. The selectivity of C_2+_ alcohols (EtOH and PrOH) could reach 49.7% with a partial current density of 397.6 mA cm^−2^ (Fig. 2f and S12†). Comparably, the FE of C_2+_ alcohols over sr-Cu was only 17.3%, and the main product was CO with an FE of 43.3%. Systematic comparisons to state-of-the-art catalysts reveal that the as-synthesized wr-Cu aerogel was a very efficient electrocatalyst for C_2+_ products, especially for the high rate production of C_2+_ alcohols (Table S1†). Furthermore, sr-Cu was annealed in a N_2_ atmosphere at 400 °C for 2 h to obtain a flat crystal surface. Fig. S13† shows that the defects decreased after annealing. The FE of CO reduced and the FE of C_2+_ products enhanced (Fig. S14†), which also indicates that the C–C coupling process was favored after the defect level of the sr-Cu catalyst was reduced by annealing. The above results suggest that the selectivity differences were due to the relative amount of defects. The defects in the Cu catalysts were the active sites for the C_1_ pathway, and the flat crystal surfaces were the active sites for C_2+_ products. In order to verify that the products were derived from CO_2_, we used isotope labeled ^13^CO_2_ or Ar to replace CO_2_ in the same set-up. From ^1^H NMR spectra in Fig. S15,† we can see the H signals of HCOOH, CH_3_COOH, EtOH and PrOH, which split into two peaks by coupling with the ^13^C atom. These results confirm that the feed gas CO_2_ was the only source of carbon in the reduction products.

We also determined the electrochemical double-layer capacitance (*C*_dl_), which was calculated from cyclic voltammogram (CV) curves to obtain the electrochemical active surface area (ECSA) of the catalysts (Fig. S16a–e†).^36^ The linear slopes in Fig. S16f† show that the C_dl_ value of sr-Cu was 23.5 mF cm^−2^, which was higher than that of wr-Cu (7.4 mF cm^−2^). This is consistent with the TEM results that sr-Cu possessed a smaller particle size than wr-Cu. After normalizing the partial current density of C_2+_ to ECSA (Fig. S17†), wr-Cu still exhibited the largest current density in the potential range from −0.6 V to −1.2 V *vs.* RHE, which indicates that the superior intrinsic CO_2_RR activity mainly originated from the surface modulation of the defect level. The results of long-term stability experiments (Fig. S18†) demonstrated that wr-Cu was stable at least for 15 h at 800 mA cm^−2^. After the continuous CO_2_ electrolysis, the morphology (Fig. S19†) and low defect crystal surface (Fig. S20†) of wr-Cu were well preserved. Moreover, the *D*_s_ value of wr-Cu was 3.25 after 15 h continuous electrolysis (Fig. S21†), which is very close to that of the initial wr-Cu (*D*_s_ = 3.28). This indicates that the Cu aerogel with porous network structures could be maintained at a high current density for long-term electrolysis due to the natural porous network structures and metallic state.

**Fig. 3:**
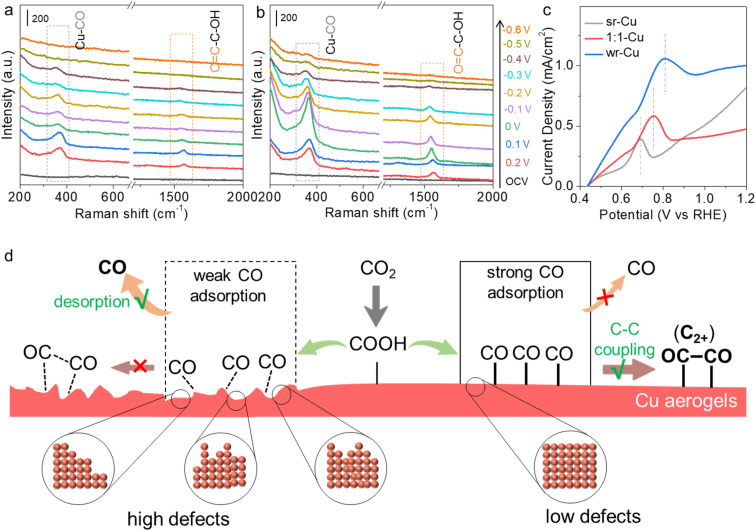
The *in situ* Raman spectra for sr-Cu (a) and wr-Cu (b) aerogels at various potentials (*vs.* RHE) during the CO_2_RR. OCV means open-circuit voltage. (c) The electrochemical CO stripping voltammetry test for sr-Cu, 1 : 1-Cu and wr-Cu aerogels in CO-saturated 0.1 M Na_2_SO_4_ solution. (d) Proposed structure–activity relationship diagram on different Cu aerogel surfaces: comparison of key steps in the C_1_ and C_2+_ reaction pathways.

Further investigation of the kinetics of the C_1_ and C_2+_ pathway on different Cu aerogels is very interesting. Therefore, an *in situ* Raman spectroscopy study was carried out over the sr-Cu and wr-Cu aerogels in a custom-built Raman setup (Fig. S22†). We could monitor the formation and dimerization of *CO to study the pathway, because it is well known that the activity and selectivity of C_2+_ products in the CO_2_RR are closely related to the formation of the *CO intermediate and subsequent dimerization on the catalyst surface.^37^ As depicted in Fig. 3a and b, there are no peaks indexed to Cu_2_O in the range of 400–650 cm^−1^, which indicated that there were no Cu^+^ species on the surfaces of sr-Cu and wr-Cu at the reduction potentials.^38^ As the applied potential shifted negatively, the peaks at 365 cm^−1^ and 1560 cm^−1^ became cognizable on both sr-Cu and wr-Cu, corresponding to the restricted rotation of Cu–CO stretching and O=C–C–OH vibration, respectively.^39,40^ The appearance of the O=C–C–OH intermediate indicates that after *CO dimerization, the oxygen atom of the CO dimer was first hydrogenated and subsequently reduced to C_2+_ products. The decrease of *CO peak intensity at the potentials more negative than 0.1 or 0 V *vs.* RHE could be attributed to the C_2+_ product process.^41^ At more negative potentials (−0.5 and −0.6 V *vs.* RHE), the band of Cu–CO disappeared over sr-Cu but it was still noticeable over wr-Cu, which indicates the stronger adsorption of *CO on the wr-Cu catalyst.^42^ Therefore, we can conclude that the wr-Cu aerogel can stabilize the *CO intermediate and promote C–C coupling for the C_2+_ pathway. Moreover, the gradual disappearance of Raman signals at more negative potentials indicates that these intermediates undergo very fast CO_2_ reduction kinetics, representing a faster reaction rate. In addition, we observed that the Raman peaks from both catalysts experienced a continuous red shift as the applied potential decreased, which could be explained by the electrochemical Stark effect, a phenomenon where the local electric field could regulate the interaction between the catalyst active centres and adsorbates, thus shifting the vibrational frequencies of intermediates.

To investigate the strength of CO binding on the different catalysts, we conducted further experiments. The electrochemical CO stripping voltammetry tests were performed to probe the CO desorption ability on the as-synthesized Cu aerogels.^43^ As shown in Fig. 3c, a sharp CO stripping profile with a dominant peak appeared around 0.81 V *vs.* RHE for wr-Cu, whereas they were around 0.69 V and 0.75 V *vs.* RHE for sr-Cu and 1 : 1-Cu, respectively. The positive shift of the peak indicates that wr-Cu has stronger binding ability towards CO, leading to higher selectivity for C_2+_ products, which is consistent with the results in the *in situ* Raman spectra. In the context of these findings, we then conducted an in-depth study of the pathway. By modulating the nucleation and growth rate of Cu using different reductants, the defect level on the Cu aerogel crystal surface could be changed, and the desorption and dimerization of *CO intermediates could be regulated to tune C_2+_ selectivity (Fig. 3d). Particularly, the strong reductant induced abundant defects in the Cu aerogel, which was beneficial to the desorption process of the *CO intermediate for the high FE of CO, whereas the low defect Cu formed with the weak reductant could not only enhance the adsorption strength of CO intermediates, but also made the adsorption direction orderly, efficiently promoting C–C coupling to C_2+_ products.

**Fig. 4:**
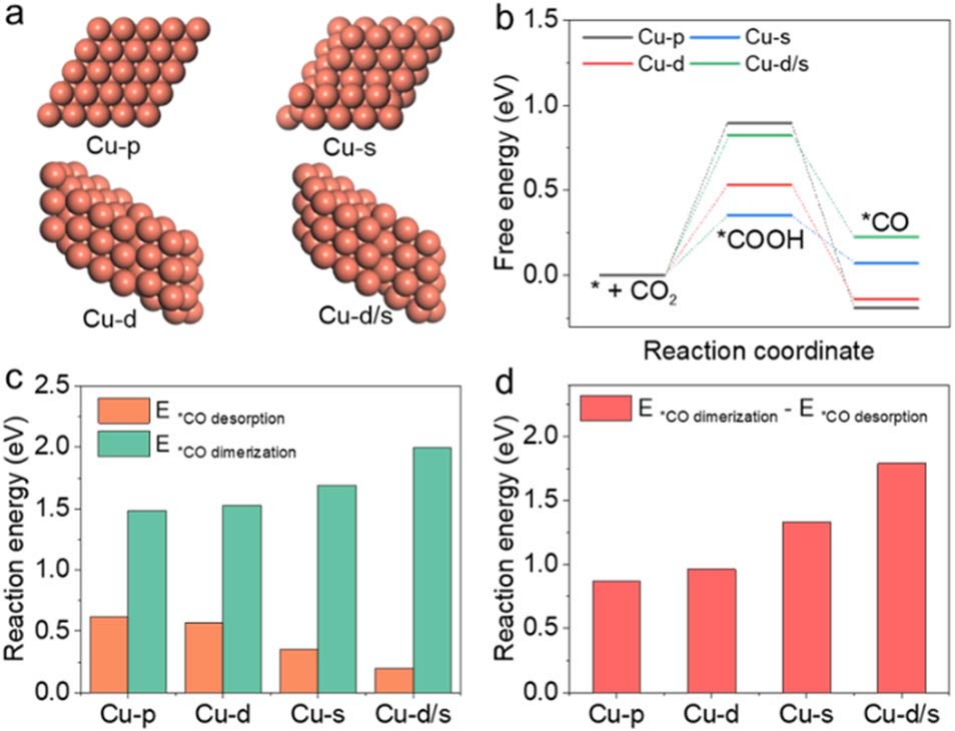
(a) DFT periodic slab models for Cu(111)-pristine, Cu(111)-dislocation, Cu(111)-step and Cu(111)-dislocation/step structures (denoted as Cu-p, Cu-d, Cu-s and Cu-ds, respectively). (b) Gibbs free-energy diagrams for the proposed steps of conversion of CO_2_ to *CO intermediate. (c) Reaction energies for *CO desorption to CO and *CO coupling to *OCCO on Cu catalysts with various defects. (d) Differences in reaction energies for *CO coupling to *OCCO and *CO desorption to CO.

It is also interesting to study how the reaction pathway varies with surface structures. In this work, density functional theory (DFT) calculations were used to study the catalytic activity for C_2+_ products on these active sites. Considering that *CO was the crucial reaction intermediate, and its adsorption/desorption strength at active sites depended on the properties of Cu surfaces, four model structures including Cu(111)-pristine, Cu(111)-dislocation, Cu(111)-step and Cu(111)-dislocation/step were selected to carry out the theoretical simulations. As shown in Fig. 4a, we denote them as Cu-p, Cu-d, Cu-s and Cu-d/s, respectively. In the CO_2_RR process, the *CO intermediate formation was subject to the hydrogenation of CO_2_ molecule (*COOH) in the aqueous electrolyte (Fig. 4b). The irregular and defective Cu structures (Cu-d, Cu-s and Cu-d/s) could reduce the reaction energies for the CO_2_ hydrogenation, which are beneficial to the subsequent formation of *CO intermediates. However, the energy for *CO desorption for CO formation was significantly reduced at the defective Cu structures, reaching 0.2 eV at the Cu-d/s (Fig. 4c). At the same time, the reaction energies associated with the dimerization of *CO intermediates toward *OCCO showed an upward trend over the defective Cu structures (Fig. S23 and S24†). We then calculated the reaction energy differences of *CO dimerization and *CO desorption over the different structures (Fig. 4d). The *CO dimerization was much easier on the Cu-p surface, while the *CO desorption was easier on the Cu-d/s surface. This further indicates that compared to Cu-d, Cu-s and Cu-d/s, the Cu-p structure could improve C_2+_ selectivity. Ultimately, our results corroborated that the CO desorption/dimerization depended on the local geometry of the surface; the defect Cu-d, Cu-s and Cu-d/s sites were responsible for CO production, while Cu-p sites favoured C_2+_ product generation; due to the low defect the Cu surface could facilitate C–C coupling.

## Conclusions

In summary, we found that crystal growth kinetics can guide the generation of efficient surface sites in Cu aerogels for increasing C_2+_ product selectivity using a room-temperature one-step synthetic strategy. The crystal growth rate is pivotal to rationalize the electrocatalytic properties of the catalysts. Using wr-Cu as the catalyst, the FE of C_2+_ products can reach 85.8% at the current density of 800 mA cm^−2^ and potential of −0.91 V *vs.* RHE, and the C_2+_ alcohol selectivity is 49.7% with a partial current density of 397.6 mA cm^−2^. The adsorption of the important *CO intermediate is notably enhanced on wr-Cu, which offers abundant precursors for C–C coupling and further reduction. Theoretical studies further suggest that the CO adsorption strength on Cu sites depends on the nature of defects. A high defect structure is beneficial for reducing the desorption energy of the *CO intermediate to get the CO product, whereas the relatively flat Cu aerogel with low defects favors the C–C coupling pathway, which leads to a high selectivity of C_2+_ products. This work provides a new route to design efficient catalysts towards C_2+_ products in the CO_2_RR, and brings new insights into the role of surface defects in electrochemistry.

## Data availability

All experimental data is available in the ESI.†

## Author contributions

P. S. L., Q. G. Z. and B. X. H. proposed the project, designed the experiments, and wrote the manuscript. P. S. L. performed the whole experiments. P. S. L., J. H. B., J. Y. L., C. J. C., X. F. S., J. L. Z. and Z. M. L. performed the analysis of experimental data. Q. G. Z. and B. X. H. co-supervised the whole project. All authors discussed the results and commented on the manuscript.

## Conflicts of interest

There are no conflicts to declare.

## Supplementary Material

SC-014-D2SC04961A-s001
