# Supplementary material for: A crystal growth kinetics guided Cu aerogel for highly efficient CO2 electrolysis to C2+ alcohols
Source: Chem Sci. 2022 Dec 6;14(2):310–6. doi: 10.1039/d2sc04961a (PMC9811509; doi:10.1039/d2sc04961a)
Supplement: SC-014-D2SC04961A-s001 [file SC-014-D2SC04961A-s001.pdf]

Electronic Supplementary Material (ESI) for Chemical Science.
This journal is © The Royal Society of Chemistry 2022

Electronic Supplementary Material (ESI) for

# Crystal growth kinetics guided Cu aerogel for highly-efficient $CO_2$ electrolysis to $C_{2+}$ alcohols

Pengsong Li[a], Jiahui Bi[a, b], Jiyuan Liu[a, b], Qinggong Zhu[a, b,*], Chunjun Chen[a], Xiaofu Sun[a, b], Jianling Zhang[a, b], Zhimin Liu[a, b] and Buxing Han[a, b, c, d,*]

[a.]Beijing National Laboratory for Molecular Sciences, CAS Key Laboratory of Colloid, Interface and Chemical Thermodynamics, CAS Research/Education Center for Excellence in Molecular Sciences, Institute of Chemistry, Chinese Academy of Sciences, Beijing100190, P. R. China

E-mail: qgzhu@iccas.ac.cn; hanbx@iccas.ac.cn

[b.]University of Chinese Academy of Sciences, Beijing 100049, P. R. China

[c.]Shanghai Key Laboratory of Green Chemistry and Chemical Processes, School of Chemistry and Molecular Engineering, East China Normal University Shanghai 200062, P. R. China.

[d.]Institute of Eco-Chongming, 20 Cuiniao Road, Chenjia Town, Chongming District, Shanghai, 202162, P. R. China.

## Methods

### Chemicals

KOH (A. R. grade), $NaBH_4$ (A. R. grade), acetone (A. R. grade) and Ni foam were provided by Sinopharm Chemical Reagent Co., Ltd, China. Copper disodium edta dihydrate (98%, Cu-EDTA) was purchased from Adamas-beta. $NH_3{\cdot}BH_3$ (A. R. grade) was obtained from Alfa Aesar China Co., Ltd. Both $CO_2$ and Ar (Beijing Beiwen Gas Chemical Industry Co., Ltd., research grade) had purity of 99.999% and used as received. Aqueous solutions were prepared with deionized water (Millipore 18.2 MΩ cm).

### Preparation of catalysts

The Cu aerogel samples were prepared through a facile one-step method using Cu-EDTA solution and $NaBH_4$ or $NH_3{\cdot}BH_3$ reductive solution as precursors. To synthesize the defect-rich Cu aerogel catalyst (sr-Cu), 5 mL solution with 2 mmol Cu-EDTA was injected rapidly into the $NaBH_4$ solution (2.5 M, 2 mL) until no bubbles formed. The precipitates obtained were subsequently washed three times with deionized water and one time with acetone. Then, the catalyst powder was immediately dried under vacuum overnight. The Cu aerogel catalyst with a relatively flat surface (wr-Cu) was fabricated by the same procedure, except for using $NH_3{\cdot}BH_3$ solution (2.5 M, 2 mL) to replace the $NaBH_4$ solution. In order to synthesize the catalysts with different defect level, different reducing solutions were obtained by mixing $NaBH_4$ and $NH_3{\cdot}BH_3$ with different molar ratios (5:1, 1:1 and 1:5), which were used to prepare 5:1-Cu, 1:1-Cu and 1:5-Cu aerogels using the same method. Before carrying out material characterizations, the Cu aerogel samples were pre-reduced under the current density of 500 mA $cm^{-2}$ for 0.5 h, and the other conditions of pre-reduction were the same as that of the $CO_2$ reduction.

**Material characterization**

The TEM characterization was carried out using a JEOL JEM-2100F. The EELS was conducted using a Thermo Fisher Scientific Themis 300. The X-ray adsorption spectroscopy (XAS) measurements were performed at 1W1B beamline station of Beijing Synchrotron Radiation Facility. The energy was tuned by Si (111) monochromator. The Cu K-edge spectra were collected in transmission mode. The as-prepared sample powder (100 mg) was directly coated on the adhesive tape (Scotch® Magic™ Tape, 1*0.5 $cm^2$) for the X-ray absorption spectroscopy collection. Powder X-ray diffraction (XRD) patterns were acquired from an X-ray diffractometer (Model D/MAX2500, Rigaka) with Cu-Kα radiation, and the scan speed was $5^{o}$ $min^{-1}$. X-ray photoelectron spectroscopy (XPS) analysis was conducted on the Thermo Scientific ESCALab 250Xi (USA) using 200 W monochromatic Al Kα radiation. The 500 μm X-ray spot was used for XPS analysis. The base pressure in the analysis chamber was about $3\times10^{-10}$ mbar. ICP-MS measurement (Thermo X Series II ICP/MS quadrupole system, Thermo Fisher Scientific) was employed to investigate the chemical composition of the Cu aerogels. In situ Raman measurements were carried out using a Horiba LabRAM HR Evolution Raman microscope in a modified flow cell, which was produced by GaossUnion (Tianjin) Photoelectric Technology Company. A 785 nm excitation laser was used and signals were recorded using a 20 s integration and by averaging two scans. The signals were recorded at different applied potential, and a 5 min electrolysis was conducted to gain the steady state before the collection of Raman spectra with constantly flowed gaseous $CO_2$. Small angle X-ray scattering (SAXS) experiments were carried out at Beamline 1W2B at the Beijing Synchrotron Radiation Facility. The data were collected using a CCD detector with maximum resolution of 981×1043 pixels. The wavelength of the X-ray was 1.41 Å, and the distance of the sample to detector was 1334.7 mm. The 2D SAXS images were obtained from the detector and then transformed into the profiles of intensity ($I(h)$) vs. wavevector ($h$) by the software FiT2D.

**Preparation of cathode electrodes**

The catalyst ink was prepared by ultrasonic dispersion of 5 mg of the catalyst powder with 20 μL Nafion solution (5 wt %) in 1 mL acetone for 30 min. Next, the as-prepared ink was drop-coated on the polytetrafluoroethylene (PTFE)-hydrophobized carbon fiber paper (Toray, YLS-30T GDL) achieving a catalyst loading of 1.0 mg $cm^{-2}$. The electrode was then dried in the atmosphere for the subsequent electrochemical testing experiments.

**Electrocatalytic $CO_2$ reduction**

Electrochemical studies were conducted in an electrochemical flow cell which included a gas chamber, a cathodic chamber, and an anodic chamber. An anion exchange membrane (FumasepFAA-3-PK-130) was used to separate the anodic and cathodic chambers, and a Hg/HgO electrode (1M KOH used as the filling solution) and Ni foam were used as the reference and counter electrodes, respectively. The electrolysis was conducted using a CHI 660e electrochemical workstation equipped with a high current amplifier CHI 680c. All potentials were converted to the RHE reference scale using the relation $E_{RHE} = E_{Hg/HgO} + 0.098 + 0.059 \times pH$ and compensated with the solution resistance. In the performance study, 1 M KOH was used as the electrolyte, and it was

circulated through the cathodic and anodic chambers using peristaltic pumps at a rate of 10 mL $min^{-1}$. The flow rate of $CO_2$ gas through the gas chamber was controlled to be 50 sccm using a digital gas flow controller.

**Double layer capacitance ($C_{dl}$) measurement**

The cyclic voltammetry measurement was conducted using the flow cell, and the other conditions were the same as that of the $CO_2$ reduction. Cyclic voltammogram measurements of the catalysts were conducted from -0.18 to -0.23 V vs. Hg/HgO with various scan rates to obtain the double layer capacitance ($C_{dl}$) of different catalysts. The $C_{dl}$ was estimated by plotting the Δj ($j_a$-$j_c$) at -0.205 V versus Hg/HgO against the scan rates, in which $j_a$ and $j_c$ are the anodic and cathodic current densities, respectively. The linear slope was equivalent to twice of the $C_{dl}$.

**Gaseous and liquid products analysis**

The gaseous product in the electrochemical experiment was collected by using a gas bag and analyzed by gas chromatography (GC, HP 4890D). The liquid products were quantified using nuclear magnetic resonance spectroscopy ($^1$H NMR). $^1$H NMR spectra of freshly acquired samples were collected on a Bruker Avance III 400 HD spectrometer. To accurately integrate the products in NMR analysis, two standards located in different regions were used in NMR analysis. The sodium 2, 2-dimethyl-2-silapentane-5-sulfonate (DSS) was the reference for acetic acid, ethanol and n-propanol, and the phenol was the reference for formate. 400 μL catholyte after the reaction was mixed with 100 μL 6 mM DSS solution, 100 μL 200 mM phenol and 200 μL $D_2O$, and then analyzed by 1H NMR.

The Faradaic efficiency (FE) of product is:

$$\boldsymbol{F}E = \frac{n}{Q / NF} \times 100\%$$

Where $Q$ is charge (C), $F$ is Faradaic constant (96485 C $mol^{-1}$), $N$ is the number of electrons required to generate the product, $n$ is the moles of products. For the $H_2$, CO, $C_2H_4$, HCOOH, $CH_3COOH$, $CH_3CH_2OH$ and n-$C_3H_7OH$, the $N$ is 2, 2, 12, 2, 8, 12 and 18, respectively.

**Theoretical calculations**

All the calculations were performed in the framework of the density functional theory with the projector augmented plane-wave method using the Vienna ab initio simulation package. [1] The generalized gradient approximation proposed by Perdew, Burke, and Ernzerh was selected for the exchange-correlation potential. [2] The long-range van der Waals interaction was described by the DFT-D3 approach. [3] The cut-off energy for plane wave was set to 400 eV. The energy criterion was set to $10^{-5}$ eV in iterative solution of the Kohn-Sham equation. A vacuum layer of 15 Å was added perpendicular to the sheet to avoid artificial interaction between periodic images. The Brillouin zone integration was performed using a 2 x 2 x 1 k-mesh. All the structures were relaxed until the residual forces on the atoms had declined to less than 0.03 eV $Å^{-1}$. The free energy was obtained from G = E + ZPE -TS + H, where E is the total energy, H is the enthalpy, S is the entropy, and ZPE is the zero-point energy at room temperature (T = 298 K). The detailed values were displayed in **Table S2**. The G(T) in **Table S2** represents ZPE -TS + H(T).

**Figures**

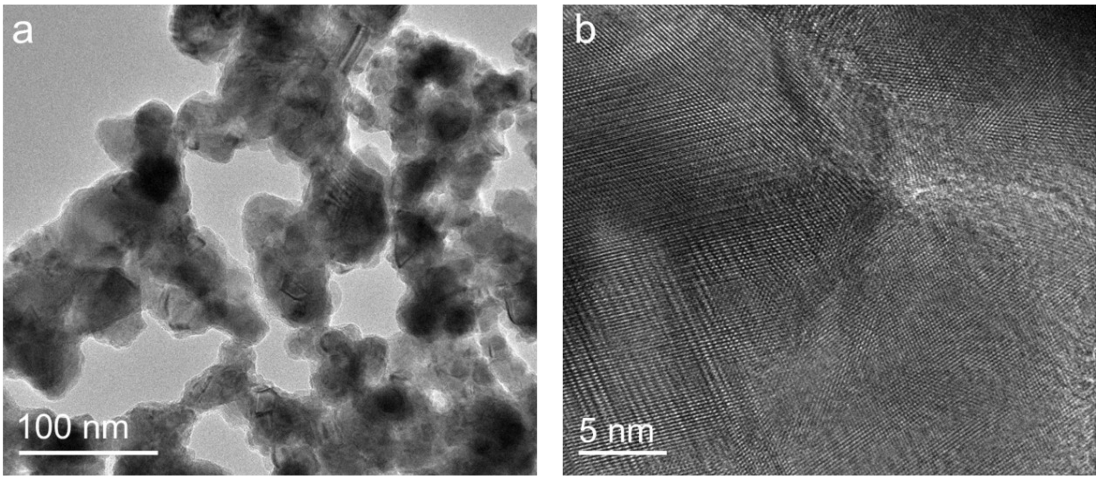


**Figure S1.** TEM and HRTEM images of 5:1-Cu aerogel.

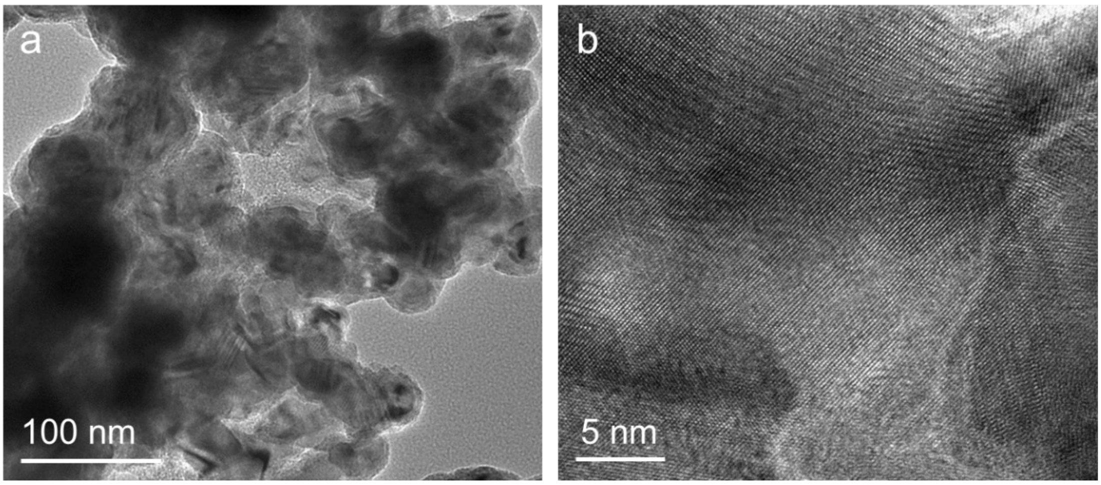


**Figure S2.** TEM and HRTEM images of 1:1-Cu aerogel.

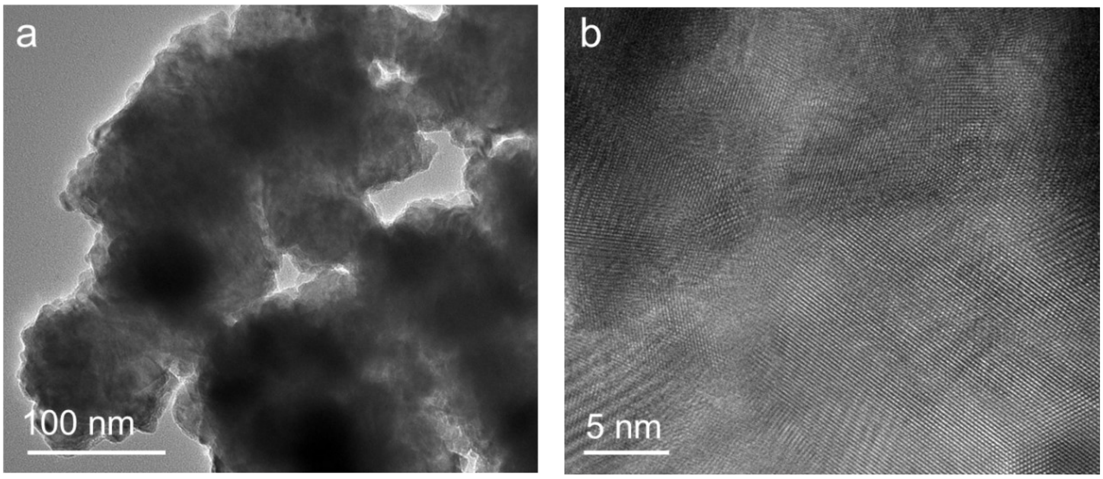


**Figure S3.** TEM and HRTEM images of 1:5-Cu aerogel.

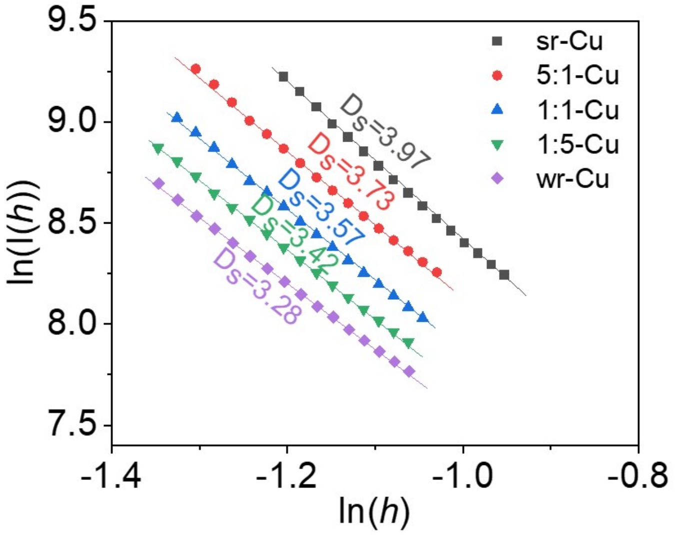


**Figure S4.** ln(I($h$)) vs. ln($h$) plots of the materials obtained from the small angle X-ray scattering data (see Figure S25). Surface fractal ($D_s$) existed in the sr-Cu, 5:1-Cu, 1:1-Cu, 1:5-Cu and wr-Cu, indicating that the crystal surface of the catalysts was defective.

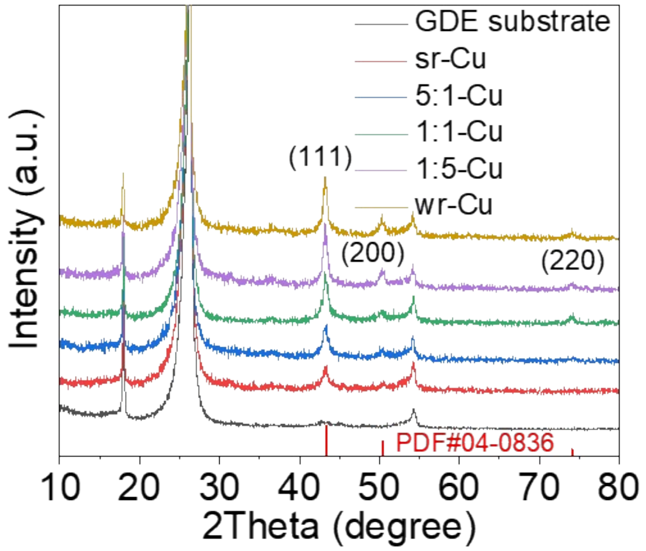


**Figure S5.** XRD patterns of sr-Cu, 5:1-Cu, 1:1-Cu, 1:5-Cu and wr-Cu aerogels after 1 h reduction electrolysis.

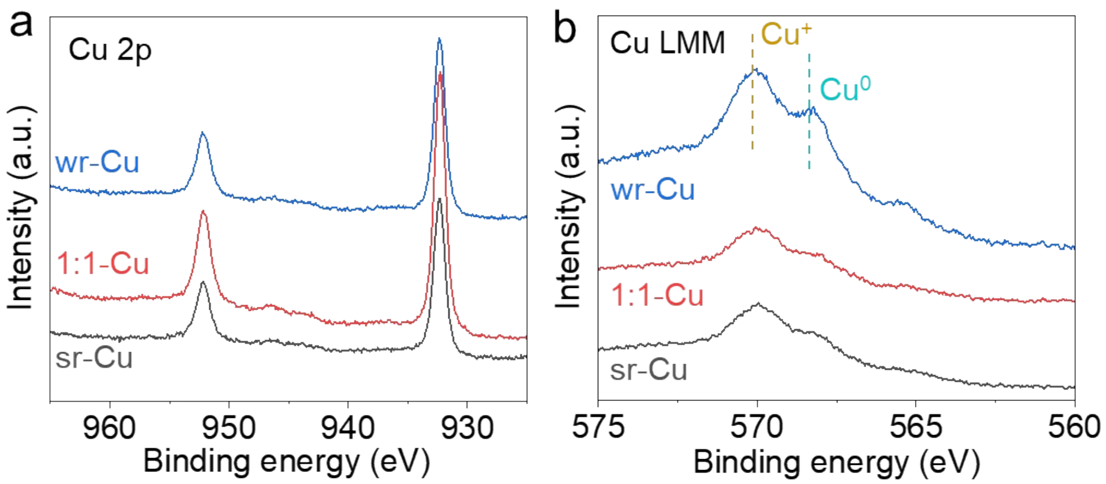


**Figure S6.** XPS spectra of Cu 2p (a) and Cu LMM (b) in the sr-Cu, 1:1-Cu and wr-Cu aerogels.

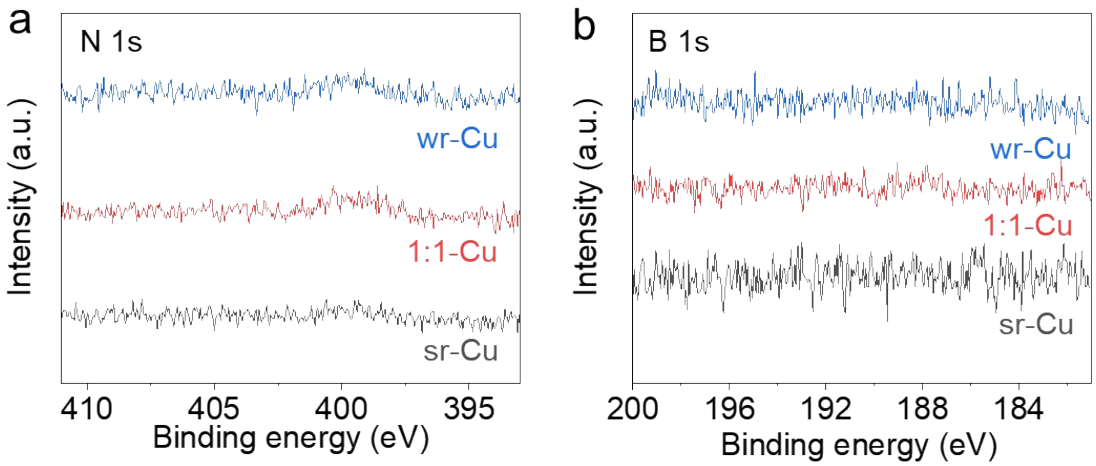


**Figure S7.** XPS spectra of N 1s (a) and B 1s (b) in the sr-Cu, 1:1-Cu and wr-Cu aerogels.

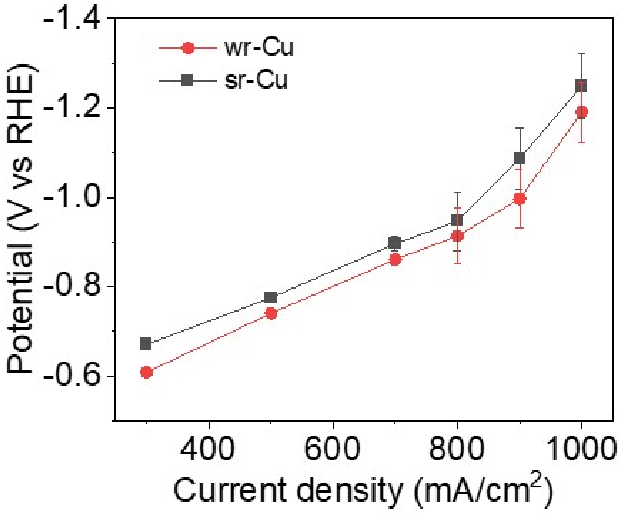


**Figure S8.** Total current densities vs. potentials over sr-Cu and wr-Cu. Error bars denote the standard deviation of potentials during the constant-current electrolysis.

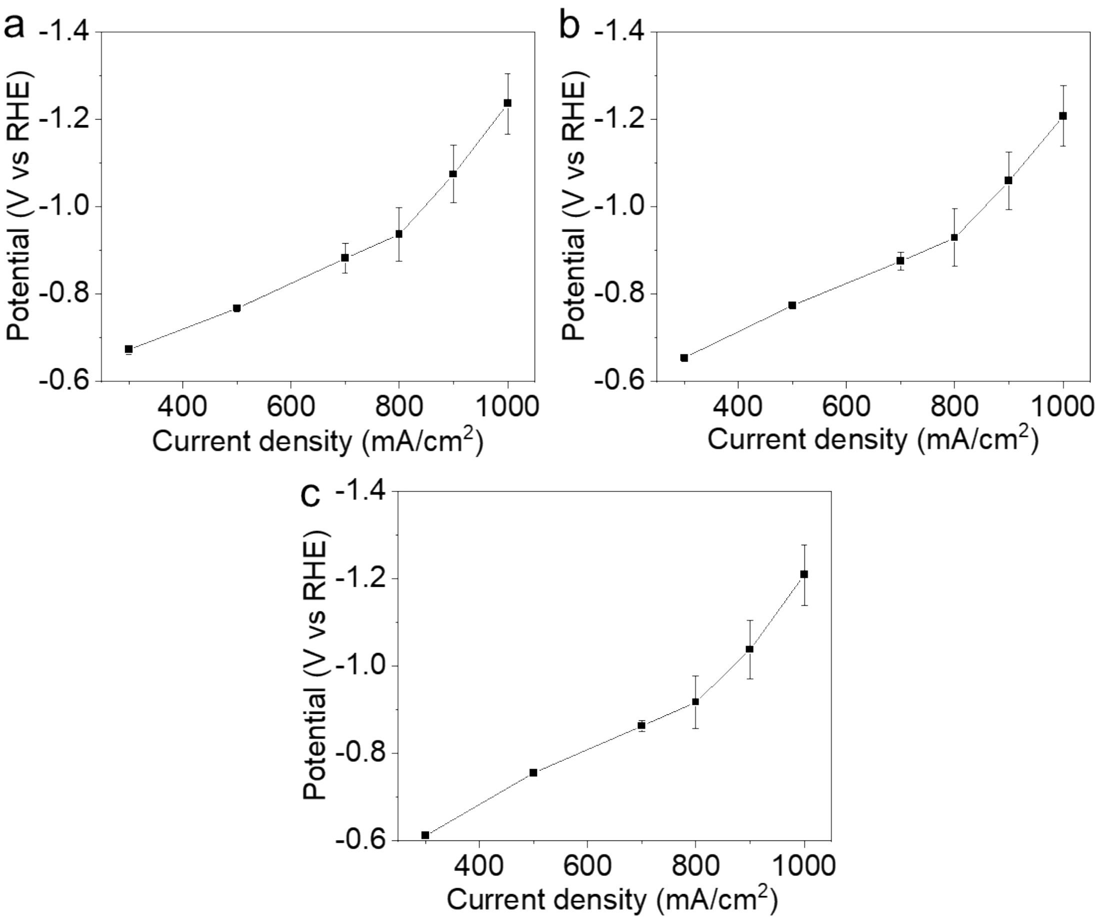


**Figure S9.** Total current densities vs. potentials of (a) 5:1-Cu, (b) 1:1-Cu and (c) 1:5-Cu aerogels. Error bars denote the standard deviation of potentials during the constant-current electrolysis.

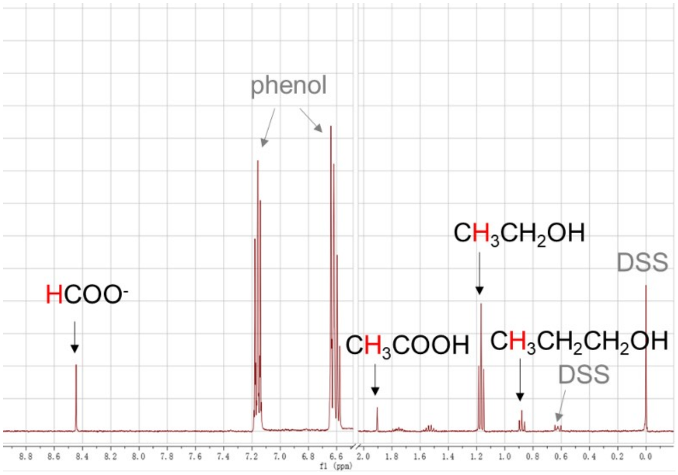


**Figure S10.** Typical $^{1}$H NMR spectra of freshly acquired liquid samples. The electrolysis experiment was carried out at the current density of 800 mA $cm^{-2}$ for 10 min using the wr-Cu aerogel as the working electrode.

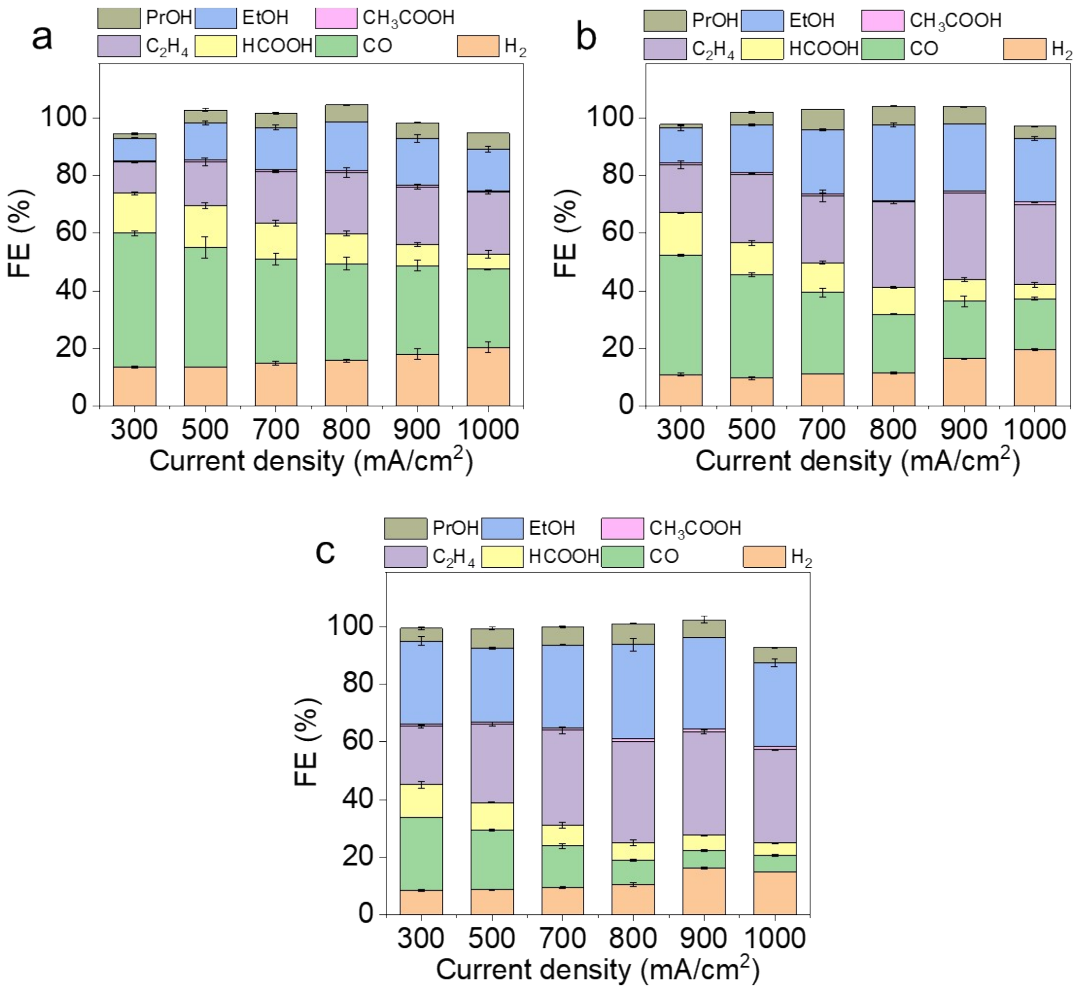


**Figure S11.** The Faradaic efficiencies (FEs) for each $CO_2$RR product and $H_2$ on (a) 5:1-Cu, (b) 1:1-Cu and (c) 1:5-Cu aerogels at various current densities ranging from 300 to 1000 mA $cm^{-2}$ in 1 M KOH. Error bars represent the standard deviations from multiple measurements.

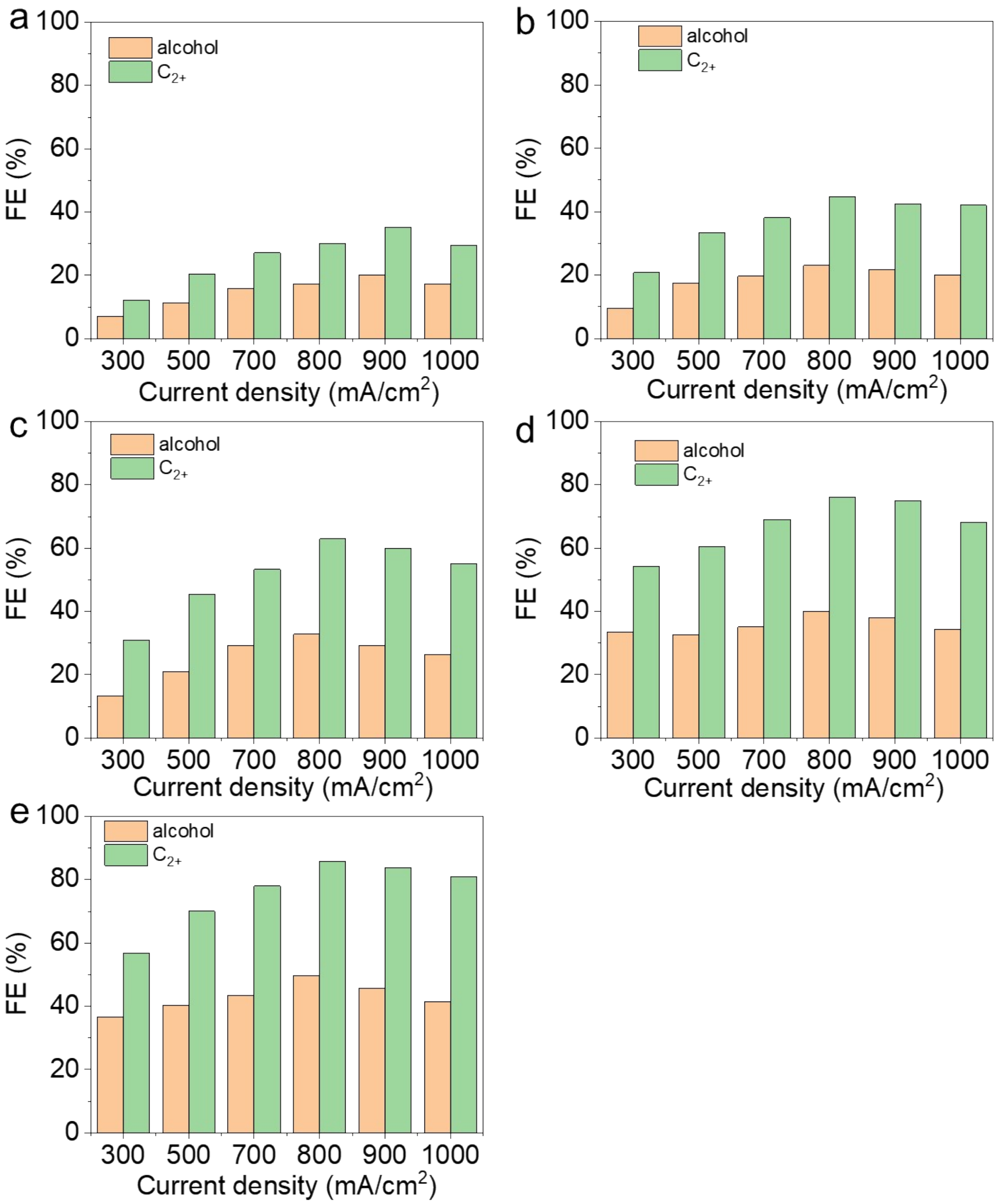


**Figure S12.** Alcohol and $C_{2+}$ FE values under different current densities over (a) sr-Cu, (b) 5:1-Cu, (c) 1:1-Cu, (d) 1:5-Cu and (e) wr-Cu aerogels.

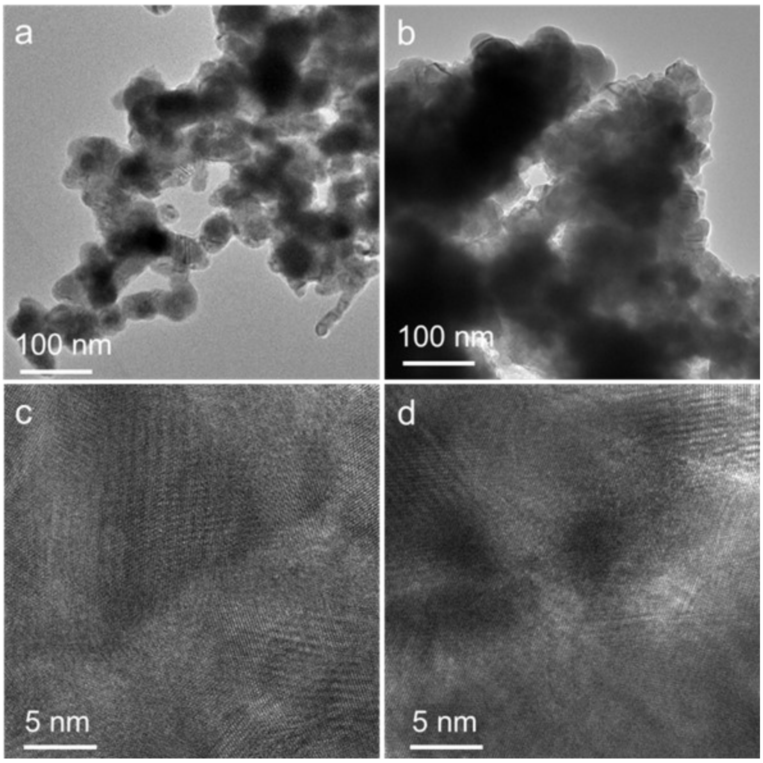


**Figure S13**. TEM and HRTEM images of sr-Cu (a, c) before and (b, d) after annealing (400 $^{O}C$, 2h, $N_2$)

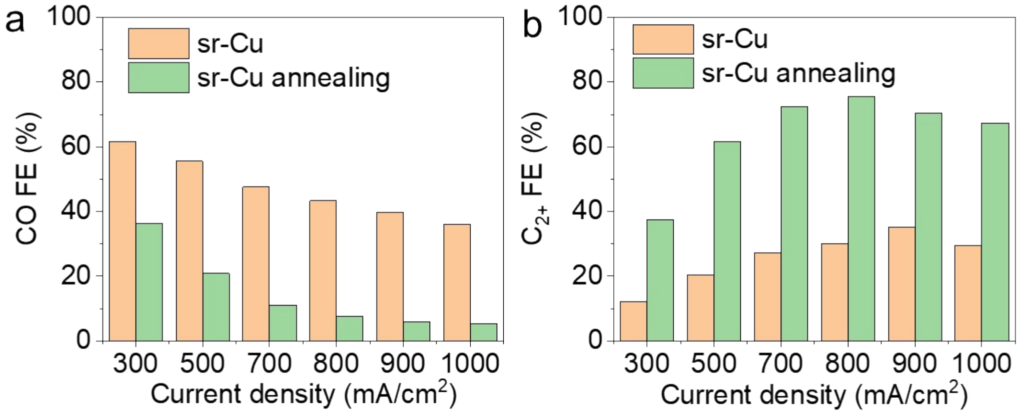


**Figure S14**. (a) CO and (b) $C_{2+}$ FE values on the sr-Cu before and after annealing (400 $^{O}C$, 2h, $N_2$) under the different current densities.

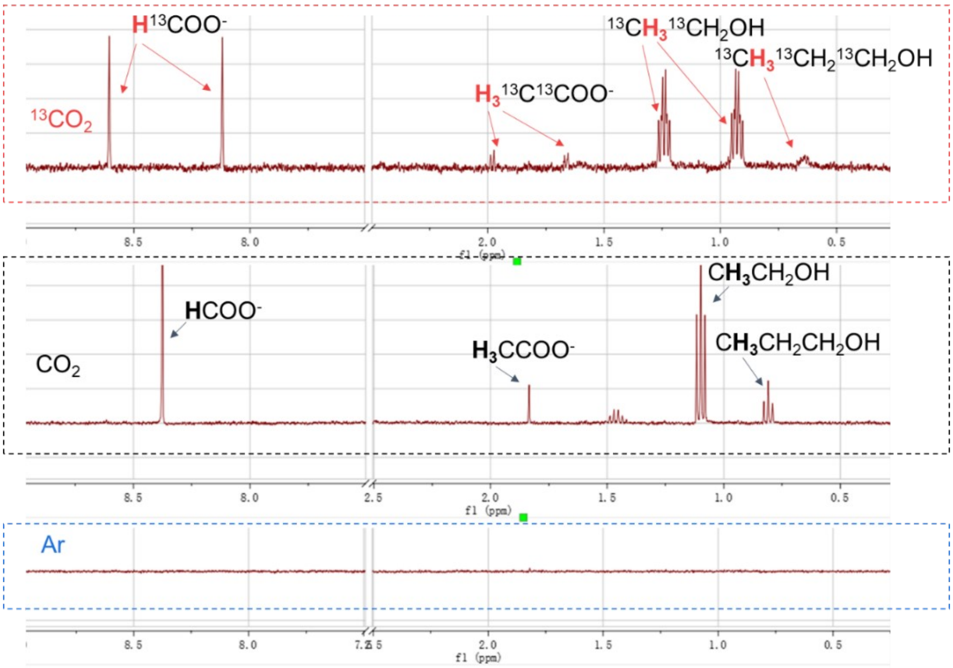


**Figure S15.** $^1$H NMR spectra of the electrolyte solutions after electrolysis using Ar, $CO_2$ and $^{13}CO_2$ as the feeding gas over wr-Cu electrode at an applied current density of 800 mA $cm^{-2}$.

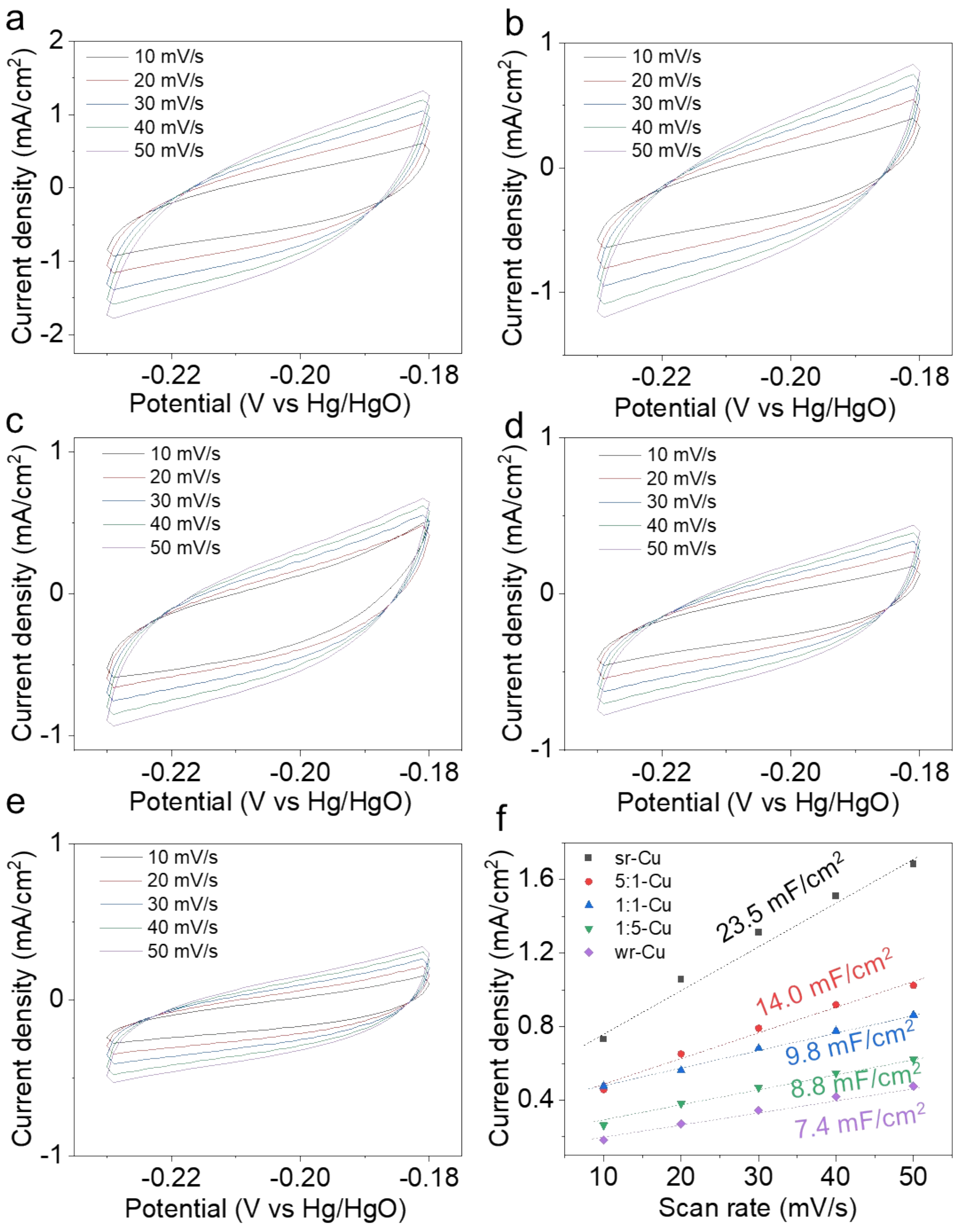


**Figure S16.** Electric double layer capacitance ($C_{dl}$) measurements at the non-Faradaic region (from -0.18 to -0.23 V vs. Hg/HgO) with various scan rates (10 mV $s^{-1}$- 50 mV $s^{-1}$) of (a) sr-Cu, (b) 5:1-Cu, (c) 1:1-Cu, (d) 1:5-Cu and (e) wr-Cu aerogels. (f) Charging current density differences plotted against scan rates.

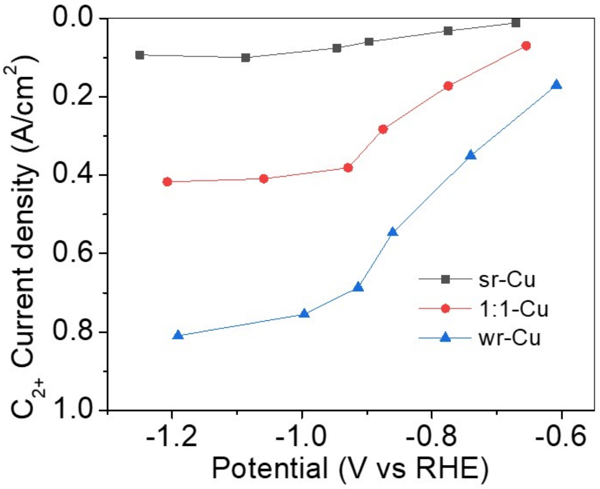


**Figure S17.** Partial current density of $C_{2+}$ is normalized to ECSA over sr-Cu, 1:1-Cu and wr-Cu. The ECAS (7.4 mF/$cm^2$) of wr-Cu was set to the unit 1 for the normalization.

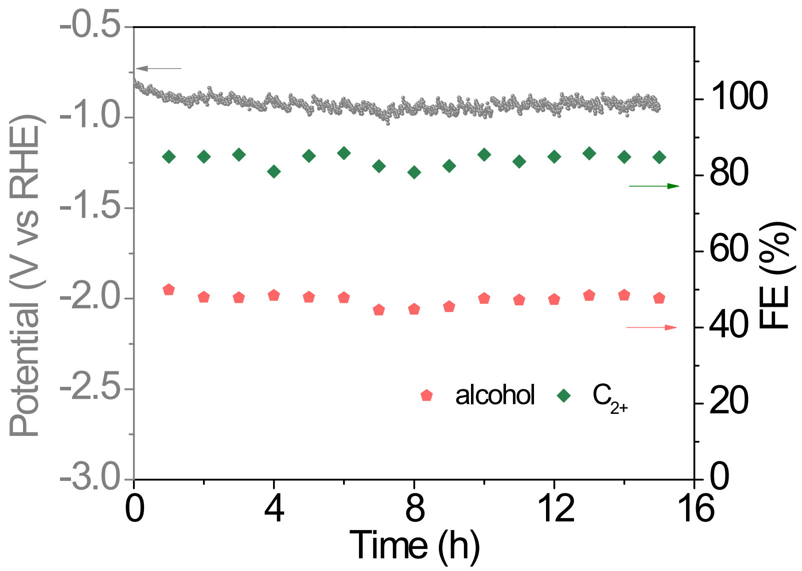


**Figure S18.** Long-term stability of wr-Cu aerogel. Electrolysis experiments were carried out at the current density of 800 mA $cm^{-2}$.

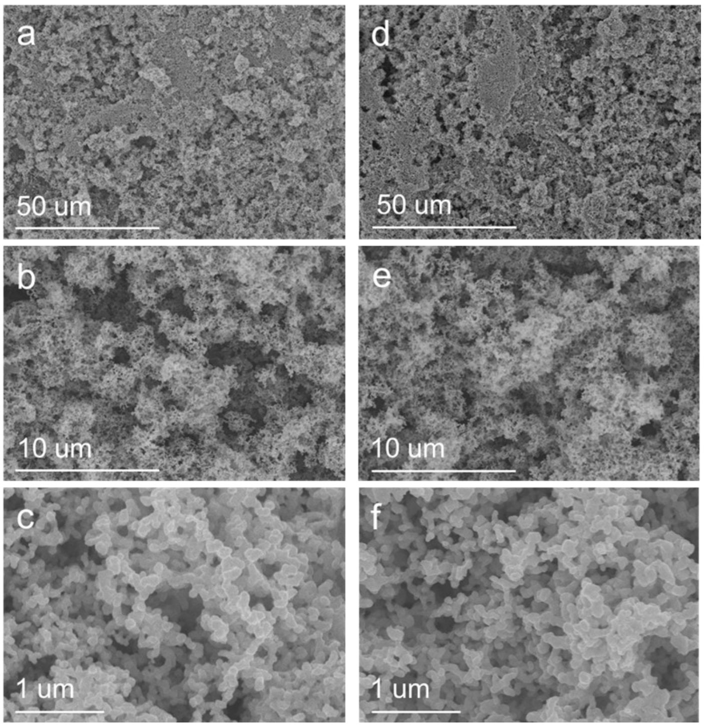


**Figure S19.** SEM images of wr-Cu aerogel electrode before (a, b, c) and after (d, e, f) 15 h continuous electrolysis with different scale bar.

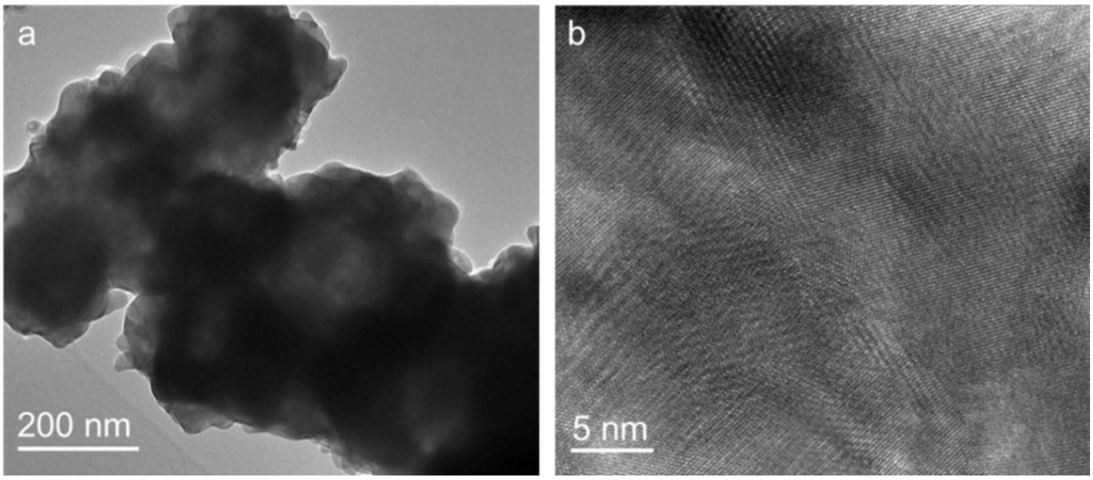


**Figure S20.** TEM and HRTEM images of wr-Cu after 15 h continuous electrolysis.

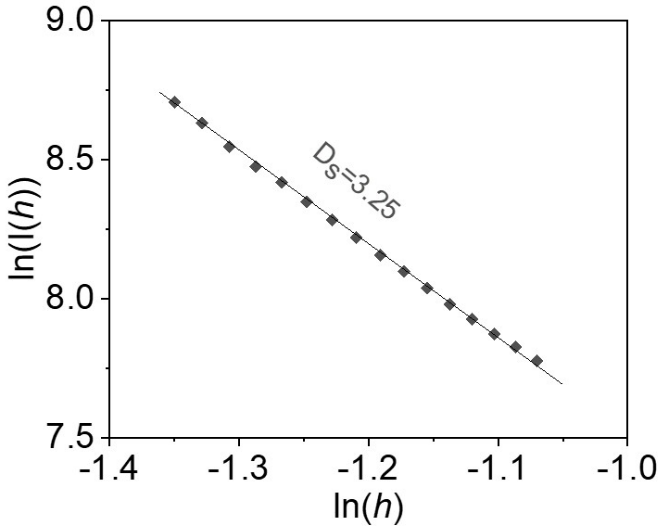


**Figure S21.** ln(**I**(*h*)) vs. ln(*h*) plots of the wr-Cu obtained from the small angle X-ray scattering data (see Figure S25) after 15 h continuous electrolysis.

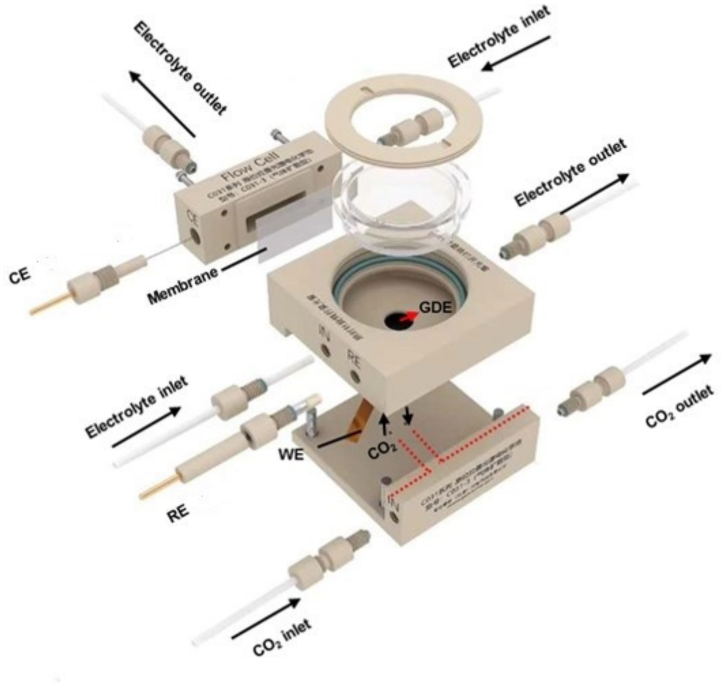


**Figure S22.** In situ electrochemical spectral cell for Raman test. CE is the counter electrode. RE is the reference electrode. WE is the working electrode.

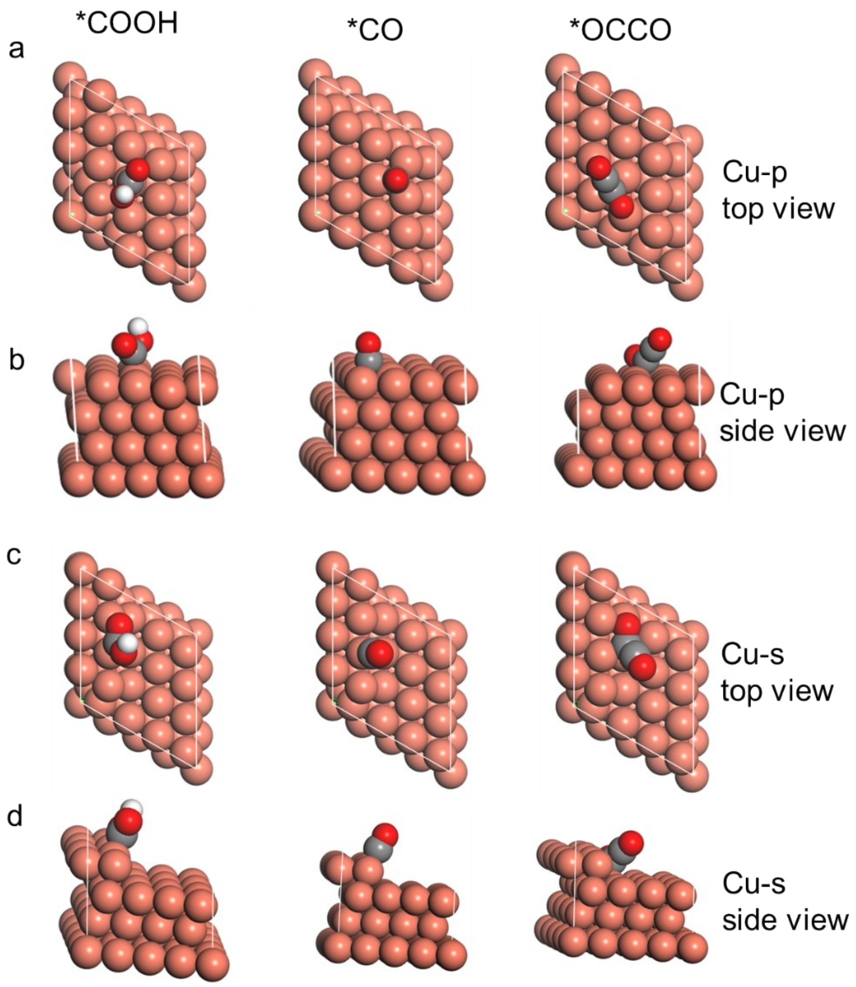


**Figure S23.** The optimized adsorption configurations of reaction intermediates on the (a, b) Cu(111)-pristine (Cu-p) and (c, d) Cu(111)-step (Cu-s) structures. The atoms in orange, grey, red and white represent Cu, C, O and H, respectively.

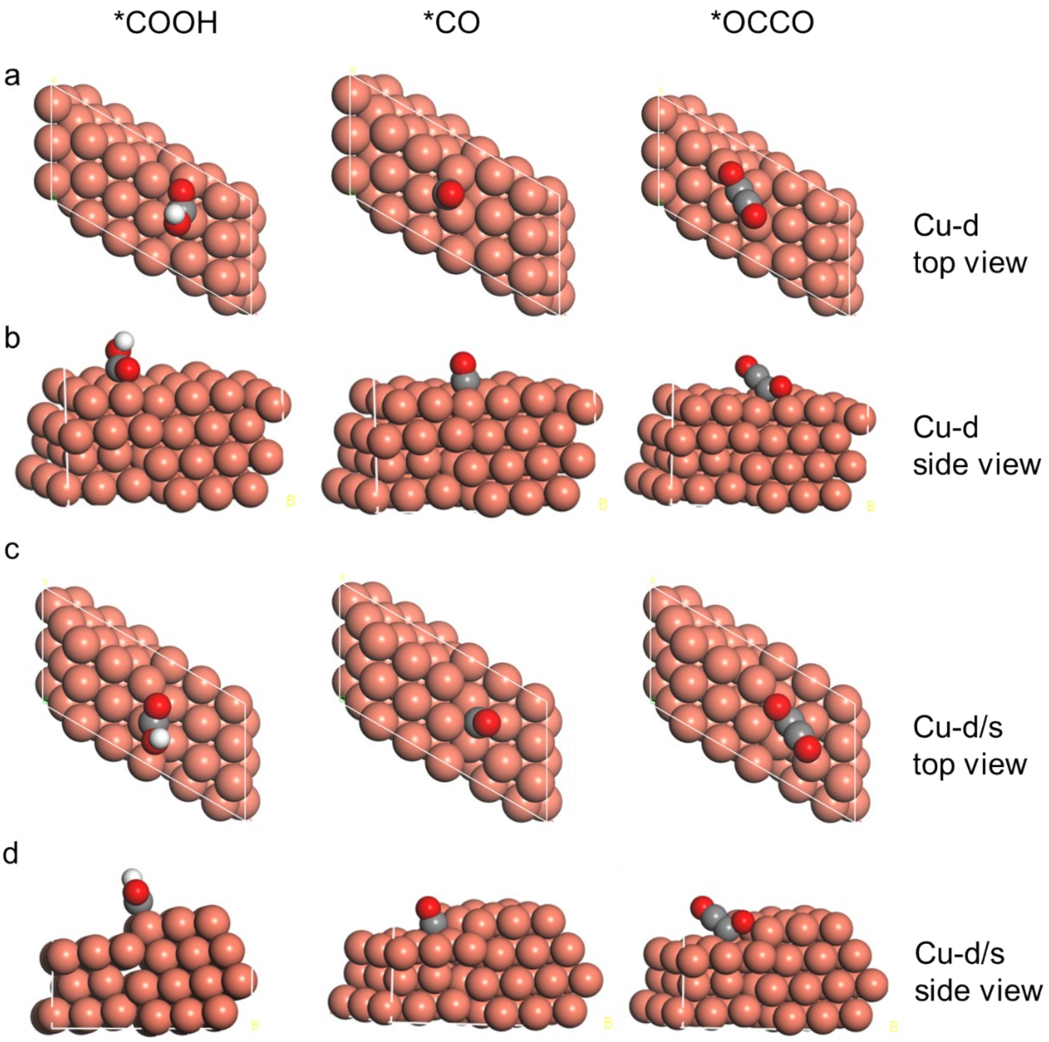


**Figure S24.** The optimized adsorption configurations of reaction intermediates on the (a, b) Cu(111)-dislocation (Cu-d) and (c, d) Cu(111)-dislocation/step (Cu-d/s) structures. The atoms in orange, grey, red and white represent Cu, C, O and H, respectively.

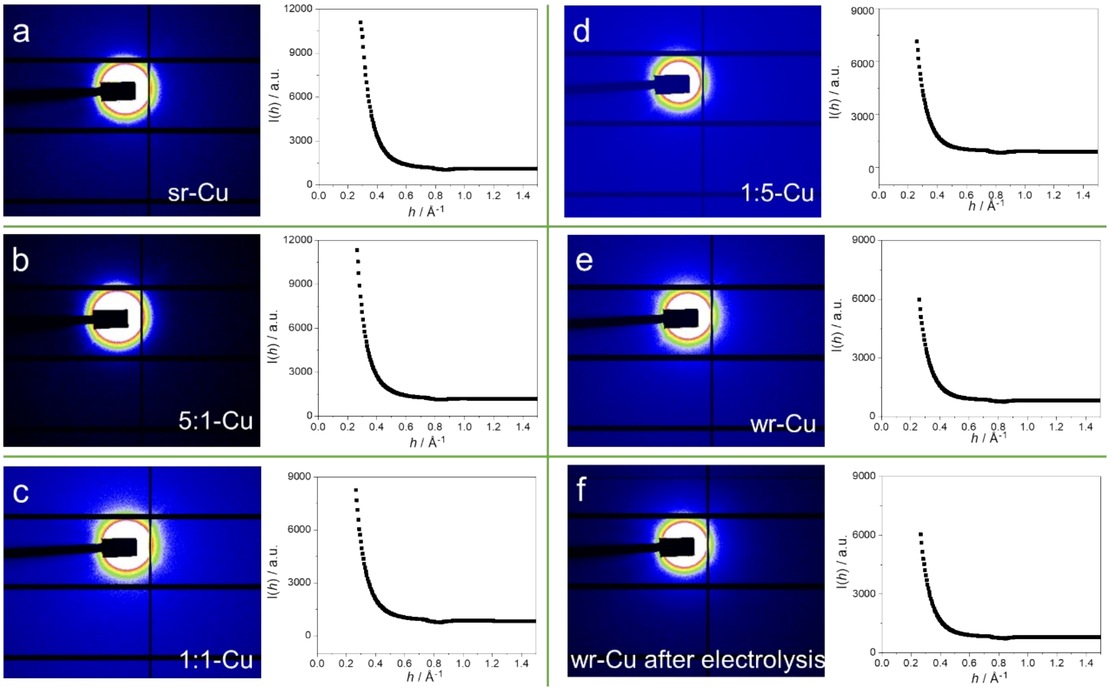


**Figure S25.** The small angle X-ray scattering data of (a) sr-Cu, (b) 5:1-Cu, (c) 1:1-Cu, (d) 1:5-Cu, (e) wr-Cu and (f) wr-Cu after electrolysis.

**Tables**

**Table S1** Comparison of FE of $C_{2+}$ products and current density (j) over as-prepared wr-Cu aerogel with some state-of-the-art catalysts in electrochemical $CO_2$ reduction.

| Catalyst | Potential (V vs RHE) | Current density (mA/cm²) | $FE_{C2+}$ (%) | $FE_{alcohol}$ (%) | $j_{C2+\ alcohols}$ (mA/cm²) | Ref. |
|---|---|---|---|---|---|---|
| **wr-Cu** | **-0.91** | **800** | **85.8** | **49.7** | **397.6** | **This work** |
| $Ag_{0.14}Cu_{0.86}$ alloy | -0.67 | 250 | 82 | 41.4 | 103.5 | 4 |
| Cu-Polyamine | -0.47 | 32 | 94 | 7 | 2.2 | 5 |
| ZnO-CuO | -0.69 | 200 | 66.7 | 46.9 | 93.8 | 6 |
| N-C/Cu[a] | -0.68 | 300 | 93.5 | 53.7 | 161.1 | 7 |
| NGQ/Cu-nr[b] | -0.9 | 282 | 80.4 | 52.4 | 147.8 | 8 |
| $Ce(OH)_x$/Cu | -0.7 | 300 | 80.3 | 43.2 | 129.6 | 9 |
| Cu(B) | -1.1 | 70 | 79 | 27 | 18.9 | 10 |
| Nanoporous Cu | -0.67 | 653 | 62 | 16.6 | 108.4 | 11 |
| CuAg wire | -0.68 | 300 | 85.9 | 25.9 | 77.7 | 12 |
| Cu-DAT[c] | -0.69 | 264 | 68.9 | 30.8 | 81.3 | 13 |
| FeTPP[Cl]/Cu[d] | -0.82 | 302 | ~85 | 41 | 123.8 | 14 |
| $Cu_2S$-Cu-V[e] | -0.92 | 400 | 55.8 | 32 | 128 | 15 |
| 100-cycle Cu | -0.963 | 68 | 60 | 27.5 | 18.7 | 16 |
| HRS-Cu[f] | -0.75 | 120 | 86.5 | 28 | 33.6 | 17 |
| N-modified Cu | -0.69 | 400 | 82.3 | 6.8 | 27.2 | 18 |
| Cu_KI 10 s | -1.09 | 40 | 72 | 19 | 7.6 | 19 |
| Cu-D[g] | -0.68 | 400 | 64 | 26.8 | 107.2 | 20 |
| 3-shell HoMSs[h] | -0.88 | 667 | 77 | ~35 | ~233.5 | 21 |
| S-HKUST-1[i] | -1.09 | 400 | 88.4 | 29.1 | 116.4 | 22 |
| B-Cu-Zn | -0.45 | 200 | 78 | 29 | 58 | 23 |
| Cu-CuI | -1.0 | ~900 | 66.1 | 33.4 | 299 | 24 |
| $Cu(OH)_2$-D[j] | -0.54 | 250 | 87 | 28 | 70 | 25 |
| multihollow $Cu_2O$ | -0.61 | 355 | 75.2 | ~30 | ~106.5 | 26 |
| (100)-Rich Cu | -0.67 | 312 | 90 | 12 | 37.4 | 27 |
| $Cu_2O$-derived Cu | N[k] | 31.2 | 59.8 | 17.2 | 5.4 | 28 |
| OD-Cu[l] | -1.0 | ~30 | 70 | ~10 | ~3 | 29 |
| CuO-derived Cu | -0.58 | 416 | 52.6 | 17.1 | 71.1 | 30 |
| Fluorine-modified Cu | -0.89 | 1600 | 80 | 13 | 208 | 31 |

[a]N-C/Cu: nitrogen-doped carbon layer on Cu, [b]NGQ/Cu-nr: nitrogen-doped graphene quantum dots on CuO-derived Cu nanorods, [c]Cu-DAT: Cu-3,5-diamino-1,2,4-triazole, [d]FeTPP[Cl]/Cu: 5,10,15,20-tetraphenyl-21H,23H-porphine iron(iii) chloride/Cu, [e]$Cu_2S$-Cu-V: $Cu_2S$-Cu-vacancy, [f]HRS-Cu: high-power reactively sputtered Cu films, [g]Cu-D: Cu dendrites, [h]3-shell HoMSs: 3-shell hollow multi-shell structures, [i]S-HKUST-1: isolated Cu-S Motifs in metal-organic framework-based pre-catalysts, [j]$Cu(OH)_2$-D: $Cu(OH)_2$-derived Cu catalyst, [k]N: no mention, [l]OD-Cu: oxide-derived copper.

**Table S2.** The correction of zero point energy (ZPE), enthalpy effect, and entropy effect of the adsorbed and gaseous species. T = 298.15 K.

| **Cu-p** | ZPE/eV | U(T)/eV | H(T)/eV | G(T)/eV | S/eV·$K^{-1}$ |
|---|---|---|---|---|---|
| $CO_2$ (gas) | 0.307 | 0.379 | 0.405 | -0.258 | 0.002 |
| *COOH | 0.585 | 0.657 | 0.657 | 0.514 | 0.0004 |
| *CO | 0.184 | 0.265 | 0.265 | 0.089 | 0.0006 |
| *OCCO | 0.386 | 0.524 | 0.524 | 0.235 | 0.0009 |
| CO (gas) | 0.131 | 0.196 | 0.222 | -0.390 | 0.002 |

| **Cu-s** | ZPE/eV | U(T)/eV | H(T)/eV | G(T)/eV | S/eV·$K^{-1}$ |
|---|---|---|---|---|---|
| $CO_2$ (gas) | 0.307 | 0.379 | 0.405 | -0.258 | 0.002 |
| *COOH | 0.609 | 0.714 | 0.714 | 0.498 | 0.0007 |
| *CO | 0.176 | 0.255 | 0.255 | 0.094 | 0.0005 |
| *OCCO | 0.396 | 0.523 | 0.523 | 0.269 | 0.0008 |
| CO (gas) | 0.131 | 0.196 | 0.222 | -0.390 | 0.002 |

| **Cu-d** | ZPE/eV | U(T)/eV | H(T)/eV | G(T)/eV | S/eV·$K^{-1}$ |
|---|---|---|---|---|---|
| $CO_2$ (gas) | 0.307 | 0.379 | 0.405 | -0.258 | 0.002 |
| *COOH | 0.604 | 0.711 | 0.711 | 0.491 | 0.0007 |
| *CO | 0.175 | 0.252 | 0.252 | 0.108 | 0.0005 |
| *OCCO | 0.386 | 0.524 | 0.524 | 0.237 | 0.0009 |
| CO (gas) | 0.131 | 0.196 | 0.222 | -0.390 | 0.002 |

| **Cu-d/s** | ZPE/eV | U(T)/eV | H(T)/eV | G(T)/eV | S/eV·$K^{-1}$ |
|---|---|---|---|---|---|
| $CO_2$ (gas) | 0.307 | 0.379 | 0.405 | -0.258 | 0.002 |
| *COOH | 0.582 | 0.656 | 0.656 | 0.510 | 0.0005 |
| *CO | 0.168 | 0.246 | 0.246 | 0.098 | 0.0005 |
| *OCCO | 0.396 | 0.523 | 0.523 | 0.266 | 0.0008 |
| CO (gas) | 0.131 | 0.196 | 0.222 | -0.390 | 0.002 |

## References


1. G. Kresse and J. Hafner, *Journal of Physics: Condensed Matter*, 1994, **6**, 8245-8257.
2. J. P. Perdew, K. Burke and M. Ernzerhof, *Phy. Rev. Lett.*, 1997, **78**, 1396-1396.
3. S. Grimme, J. Antony, S. Ehrlich and H. Krieg, *J. Chem. Phy.*, 2010, **132**, 154104.
4. Y. C. Li, Z. Wang, T. Yuan, D.-H. Nam, M. Luo, J. Wicks, B. Chen, J. Li, F. Li, F. P. G. de Arquer, Y. Wang, C.-T. Dinh, O. Voznyy, D. Sinton and E. H. Sargent, *J. Am. Chem. Soc.*, 2019, **141**, 8584-8591.
5. X. Chen, J. Chen, N. M. Alghoraibi, D. A. Henckel, R. Zhang, U. O. Nwabara, K. E. Madsen, P. J. A. Kenis, S. C. Zimmerman and A. A. Gewirth, *Nat. Catal.*, 2021, **4**, 20-27.
6. D. Ren, J. Gao, L. Pan, Z. Wang, J. Luo, S. M. Zakeeruddin, A. Hagfeldt and M. Grätzel, *Angew. Chem. Int. Ed.*, 2019, **58**, 15036-15040.
7. X. Wang, Z. Wang, F. P. García de Arquer, C.-T. Dinh, A. Ozden, Y. C. Li, D.-H. Nam, J. Li, Y.-S. Liu, J. Wicks, Z. Chen, M. Chi, B. Chen, Y. Wang, J. Tam, J. Y. Howe, A. Proppe, P. Todorović, F. Li, T.-T. Zhuang, C. M. Gabardo, A. R. Kirmani, C. McCallum, S.-F. Hung, Y. Lum, M. Luo, Y. Min, A. Xu, C. P. O'Brien, B. Stephen, B. Sun, A. H. Ip, L. J. Richter, S. O. Kelley, D. Sinton and E. H. Sargent, *Nat. Energy*, 2020, **5**, 478-486.
8. C. Chen, X. Yan, S. Liu, Y. Wu, Q. Wan, X. Sun, Q. Zhu, H. Liu, J. Ma, L. Zheng, H. Wu and B. Han, *Angew. Chem. Int. Ed.*, 2020, **59**, 16459-16464.
9. M. Luo, Z. Wang, Y. C. Li, J. Li, F. Li, Y. Lum, D.-H. Nam, B. Chen, J. Wicks, A. Xu, T. Zhuang, W. R. Leow, X. Wang, C.-T. Dinh, Y. Wang, Y. Wang, D. Sinton and E. H. Sargent, *Nat. Commun.*, 2019, **10**, 5814.
10. Y. Zhou, F. Che, M. Liu, C. Zou, Z. Liang, P. De Luna, H. Yuan, J. Li, Z. Wang, H. Xie, H. Li, P. Chen, E. Bladt, R. Quintero-Bermudez, T.-K. Sham, S. Bals, J. Hofkens, D. Sinton, G. Chen and E. H. Sargent, *Nat. Chem.*, 2018, **10**, 974-980.
11. J.-J. Lv, M. Jouny, W. Luc, W. Zhu, J.-J. Zhu and F. Jiao, *Adv. Mater.*, 2018, **30**, 1803111.
12. T. T. H. Hoang, S. Verma, S. Ma, T. T. Fister, J. Timoshenko, A. I. Frenkel, P. J. A. Kenis and A. A. Gewirth, *J. Am. Chem. Soc.*, 2018, **140**, 5791-5797.
13. T. T. H. Hoang, S. Ma, J. I. Gold, P. J. A. Kenis and A. A. Gewirth, *ACS Catalysis*, 2017, **7**, 3313-3321.
14. F. Li, Y. C. Li, Z. Wang, J. Li, D.-H. Nam, Y. Lum, M. Luo, X. Wang, A. Ozden, S.-F. Hung, B. Chen, Y. Wang, J. Wicks, Y. Xu, Y. Li, C. M. Gabardo, C.-T. Dinh, Y. Wang, T.-T. Zhuang, D. Sinton and E. H. Sargent, *Nat. Catal.*, 2020, **3**, 75-82.
15. T.-T. Zhuang, Z.-Q. Liang, A. Seifitokaldani, Y. Li, P. De Luna, T. Burdyny, F. Che, F. Meng, Y. Min, R. Quintero-Bermudez, C. T. Dinh, Y. Pang, M. Zhong, B. Zhang, J. Li, P.-N. Chen, X.-L. Zheng, H. Liang, W.-N. Ge, B.-J. Ye, D. Sinton, S.-H. Yu and E. H. Sargent, *Nat. Catal.*, 2018, **1**, 421-428.
16. K. Jiang, R. B. Sandberg, A. J. Akey, X. Liu, D. C. Bell, J. K. Nørskov, K. Chan and H. Wang, *Nat. Catal.*, 2018, **1**, 111-119.
17. G. Zhang, Z.-J. Zhao, D. Cheng, H. Li, J. Yu, Q. Wang, H. Gao, J. Guo, H. Wang, G. A. Ozin, T. Wang and J. Gong, *Nat. Commun.*, 2021, **12**, 5745.
18. J.-Y. Kim, D. Hong, J.-C. Lee, H. G. Kim, S. Lee, S. Shin, B. Kim, H. Lee, M. Kim, J. Oh, G.-D. Lee, D.-H. Nam and Y.-C. Joo, *Nat. Commun.*, 2021, **12**, 3765.
19. T. Kim and G. T. R. Palmore, *Nat. Commun.*, 2020, **11**, 3622.

20. Z.-Z. Niu, F.-Y. Gao, X.-L. Zhang, P.-P. Yang, R. Liu, L.-P. Chi, Z.-Z. Wu, S. Qin, X. Yu and M.-R. Gao, *J. Am. Chem. Soc.*, 2021, **143**, 8011-8021.
21. C. Liu, M. Zhang, J. Li, W. Xue, T. Zheng, C. Xia and J. Zeng, *Angew. Chem. Int. Ed.*, 2022, **61**, e202113498.
22. C. F. Wen, M. Zhou, P. F. Liu, Y. Liu, X. Wu, F. Mao, S. Dai, B. Xu, X. L. Wang, Z. Jiang, P. Hu, S. Yang, H. F. Wang and H. G. Yang, *Angew. Chem. Int. Ed.*, 2022, **61**, e202111700.
23. Y. Song, J. R. C. Junqueira, N. Sikdar, D. Öhl, S. Dieckhöfer, T. Quast, S. Seisel, J. Masa, C. Andronescu and W. Schuhmann, *Angew. Chem. Int. Ed.*, 2021, **60**, 9135-9141.
24. H. Li, T. Liu, P. Wei, L. Lin, D. Gao, G. Wang and X. Bao, *Angew. Chem. Int. Ed.*, 2021, **60**, 14329-14333.
25. D. Zhong, Z.-J. Zhao, Q. Zhao, D. Cheng, B. Liu, G. Zhang, W. Deng, H. Dong, L. Zhang, J. Li, J. Li and J. Gong, *Angew. Chem. Int. Ed.*, 2021, **60**, 4879-4885.
26. P.-P. Yang, X.-L. Zhang, F.-Y. Gao, Y.-R. Zheng, Z.-Z. Niu, X. Yu, R. Liu, Z.-Z. Wu, S. Qin, L.-P. Chi, Y. Duan, T. Ma, X.-S. Zheng, J.-F. Zhu, H.-J. Wang, M.-R. Gao and S.-H. Yu, *J. Am. Chem. Soc.*, 2020, **142**, 6400-6408.
27. Y. Wang, Z. Wang, C.-T. Dinh, J. Li, A. Ozden, M. Golam Kibria, A. Seifitokaldani, C.-S. Tan, C. M. Gabardo, M. Luo, H. Zhou, F. Li, Y. Lum, C. McCallum, Y. Xu, M. Liu, A. Proppe, A. Johnston, P. Todorovic, T.-T. Zhuang, D. Sinton, S. O. Kelley and E. H. Sargent, *Nat. Catal.*, 2020, **3**, 98-106.
28. A. D. Handoko, C. W. Ong, Y. Huang, Z. G. Lee, L. Lin, G. B. Panetti and B. S. Yeo, *The Journal of Physical Chemistry C*, 2016, **120**, 20058-20067.
29. Y. Lum, B. Yue, P. Lobaccaro, A. T. Bell and J. W. Ager, *The Journal of Physical Chemistry C*, 2017, **121**, 14191-14203.
30. L. R. L. Ting, R. García-Muelas, A. J. Martín, F. L. P. Veenstra, S. T.-J. Chen, Y. Peng, E. Y. X. Per, S. Pablo-García, N. López, J. Pérez-Ramírez and B. S. Yeo, *Angewandte Chemie International Edition*, 2020, **59**, 21072-21079.
31. W. Ma, S. Xie, T. Liu, Q. Fan, J. Ye, F. Sun, Z. Jiang, Q. Zhang, J. Cheng and Y. Wang, *Nat. Catal.*, 2020, **3**, 478-487.
